# Single-cell analysis of mesenchymal cells in permeable neural vasculature reveals novel diverse subpopulations of fibroblasts

**DOI:** 10.1186/s12987-024-00535-7

**Published:** 2024-04-05

**Authors:** William E. Bastedo, R. Wilder Scott, Martin Arostegui, T. Michael Underhill

**Affiliations:** 1 Department of Cellular and Physiological Sciences, University of British Columbia, 2222 Health Sciences Mall, Vancouver, BC V6T 1Z3 Canada; 2 School of Biomedical Engineering and the Biomedical Research Centre, University of British Columbia, 2222 Health Sciences Mall, Vancouver, BC V6T 1Z3 Canada

**Keywords:** Mesenchymal cells, Pituitary, Choroid plexus, Meninges, *Hic1*, Pericytes, Fibroblasts, scRNA-seq, Neurovascular, Blood brain barrier

## Abstract

**Background:**

In the choroid plexus and pituitary gland, vasculature is known to have a permeable, fenestrated phenotype which allows for the free passage of molecules in contrast to the blood brain barrier observed in the rest of the CNS. The endothelium of these compartments, along with secretory, neural-lineage cells (choroid epithelium and pituitary endocrine cells) have been studied in detail, but less attention has been given to the perivascular mesenchymal cells of these compartments.

**Methods:**

The *Hic1*^*CreERT2*^* Rosa26*^*LSL−TdTomato*^ mouse model was used in conjunction with a *Pdgfra*^*H2B−EGFP*^ mouse model to examine mesenchymal cells, which can be subdivided into Pdgfra^+^ fibroblasts and Pdgfra^−^ pericytes within the choroid plexus (CP) and pituitary gland (PG), by histological, immunofluorescence staining and single-cell RNA-sequencing analyses.

**Results:**

We found that both CP and PG possess substantial populations of distinct Hic1^+^ mesenchymal cells, including an abundance of Pdgfra^+^ fibroblasts. Within the pituitary, we identified distinct subpopulations of Hic1^+^ fibroblasts in the glandular anterior pituitary and the neurosecretory posterior pituitary. We also identified multiple distinct markers of CP, PG, and the meningeal mesenchymal compartment, including alkaline phosphatase, indole-n-methyltransferase and CD34.

**Conclusions:**

Novel, distinct subpopulations of mesenchymal cells can be found in permeable vascular interfaces, including the CP, PG, and meninges, and make distinct contributions to both organs through the production of structural proteins, enzymes, transporters, and trophic molecules.

**Supplementary Information:**

The online version contains supplementary material available at 10.1186/s12987-024-00535-7.

## Introduction

The blood–brain barrier (BBB) is a term used to encompass the distinct properties of brain vasculature. Unlike vessels in the rest of the body, the BBB is typically impermeable to macromolecules and immune cells, and is engaged in a host of active efflux transport processes [1]. The BBB is also a major obstacle to treatment, since it prevents the passage of many drug molecules, including most macromolecular biologics into the central nervous system (CNS) [2]. Not all regions of the brain have such a barrier, however, with permeable regions in the choroid plexus (CP), pituitary gland (PG), as well as smaller circumventricular organs (CVO) such as the area postrema (AP). The study of vasculature within these regions first emerged in the early 1900s in response to the discovery of vital dyes such as trypan blue which did not enter the brain outside of these specialized areas [3]. Recent investigations reported that WNT/beta-catenin signaling contributes to the BBB phenotype and is downregulated in permeable neurovascular areas [4]. In contrast, permeability of the pituitary gland is mediated, at least in part, by a combination of heightened levels of transforming growth factor beta (TGF-beta) and vascular endothelial growth factor (VEGF), as well as reduced levels of retinoic acid (RA) mediated by CYP26B1 activity [5]. However, in contrast to the extensive attention paid to the vascular endothelium of these regions, less information is available on the nature of the mesenchymal cells within these parts of the brain. Study of mesenchymal cells has been confounded by a lack of specific markers; in vivo markers of mesenchymal cells used in other tissues, such as leptin receptor (*Lepr*) [6], *Pdgfra* [7], *Prrx1* [8], *Cspg4* [9, 10] and *Nestin* [11], are nonspecific within the brain, where they label neurons and glial lineages [12].

The choroid plexus (CP) is the predominant source of cerebrospinal fluid (CSF) in the brain [13]. The CP also serves as a physiological filter, secreting neurotrophic factors to support neurogenesis [14] and functions as a drainage system for potentially harmful products such as beta amyloid [15], and serotonin [16]. Along with these roles, the CP is known to regulate the neuro-immune status of the brain by mediating neuroinflammatory signaling [17], and providing a site for immune cell entry into the brain [18]. The CP is made up of two main components: (1) a sheet of epithelial cells, which form tight junctions contributing to an impermeable ‘blood-CSF barrier’, and (2) a stromal core made up of distinct fenestrated endothelium [19], an array of immune cell subpopulations [20] and mesenchymal cells. Herein, the designation “mesenchymal cell” is being used as an umbrella term to encompass cells with mesenchymal-lineage potential including but not limited to, fibroblasts, connective tissue cells, and mural cells (pericytes and vascular smooth muscle cells, vSMCs).

Studies in the contribution of CP mesenchymal cells to CP development have been similarly restricted by the lack of effective mesenchymal cell markers [21]. As a result, most studies of CP at the cellular level have focused on the specific contributions of secretory cells and endothelium. A recent single-cell survey of all CP cell types highlighted the importance of CP pericytes, where the authors found and validated an abundance of the SARS-CoV-2 receptor ACE2 [22]. The PG, along with the CP, is a permeable region of the brain, with its selectively permeable properties first identified in early vital dye experiments [23]. Cellular diversity in the PG is somewhat better documented than it is in the CP, with a variety of recent single-cell studies investigating distinctions between the various endocrine cell types (such as somatotropes, corticotropes, gonadotropes, etc.), revealing distinct populations of endothelium, folliculostellate cells and mesenchymal subpopulations across mammals [24, 25]. Mesenchymal cells and associated matrix in the PG form a mesh-like structure, including both vessel- and non-vessel associated cells, that resembles, but is distinct from the network formed by folliculostellate cells [26]. The nature of PG mesenchyme and its contribution to barrier function is also better characterized than mesenchymal cells of the CP. For example, folliculostellate cells produce TGFb2 signals which promote collagen synthesis in PG mesenchyme in a TGFBR1-dependent manner [27]. Furthermore, the mesenchymal marker *Prrx1* has been used to demonstrate the colonization of the mouse pituitary gland by a population of fibroblasts between embryonic day 13.5 and 18.5 [28, 29]. However, a lack of specific markers (particularly between PG mesenchyme and folliculostellate cells) has hampered their further study. Herein, a labelling strategy employing the *Hic1*^*CreERT2*^ mouse line, coupled with single cell RNA-sequencing (scRNA-seq) and histological validation was used to identify and characterize mesenchymal cells within the CP and PG.

*Hypermethylated in cancer 1* (HIC1), which encodes a zinc finger transcription factor, was first identified as a candidate tumor suppressor gene based, in part, on the observation that it is often silenced in a variety of cancers and heterologous expression of HIC1 in tumor cell lines reduced colony formation [30]. Subsequent studies have shown that HIC1 contains a BTB-POZ domain and five Krüppel-like C_2_H_2_ zinc finger domains and functions primarily as a transcriptional repressor [31]. Several putative target genes have been identified, some of which regulate the cell cycle (i.e., *Cdkn1a*, *Ccnd1*) [32]. Grimm et al. showed that in the murine embryo, *Hic1* transcripts were predominantly localized to mesenchymal cells [33]. Furthermore, *Hic1* null mutants are embryonic lethal and present with a spectrum of deficiencies reflective of a broad impact on ontogenesis [34, 35]. Analysis of *Hic1* expression further revealed that in mouse embryonic fibroblasts, *Hic1* is regulated by retinoic acid (RA) signaling through RA receptor-mediated recruitment of a TET-TDG complex that leads to active DNA demethylation of the *Hic1* promoter [36]. Genetic tools based on *Hic1* were generated and used to study Hic1^+^ cells in vivo [37, 38]. In skeletal muscle, *Hic1* efficiently identified various mesenchymal cells including fibroblasts (*Pdgfra*, *Col15a1*, *Pi16*, *Dpt*), pericytes (*Rgs5*, *Kcnj8*, *Abcc9*) and specialized mesenchymal cells including Scx^+^ tenogenic and myotenogenic progenitors [38]. Further studies conducted with this mouse model have reported selective labelling of similar populations of mesenchymal cells in heart [39], skin [40] and spinal dura [41, 42]. In these studies, deletion of *Hic1* was shown to cause mesenchymal cell hyperplasia, consistent with a role for HIC1 in regulating mesenchymal cell quiescence [38–40]; this is also congruent with earlier observations in which *HIC1* expression impaired colony formation. In the developing murine embryo, *Hic1* first appears around embryonic day 9.5, and is effective at identifying a constellation of mesenchymal cells in the developing limb bud [35, 43].

Analysis of Hic1^+^ populations using the *Hic1*^*CreERT2*^ mouse line enabled specific identification of subpopulations of perivascular mesenchymal cells decorating vessels and fibrous structures that were unique to the CP, PG, and meninges, respectively. Single-cell datasets validated by immunofluorescence and histology confirm that these novel subpopulations express an abundance of genes which have been previously attributed to other cell types in the CP and PG.

## Methods

### Animals

The *Hic1*^*CreERT2*^ knock-in mice were generated using standard gene targeting methodology as described previously [38]. For labelling experiments, *Hic1*^*CreERT2*^ mice were interbred with *Rosa26*^*LSL−tdTomato*^ mice (Ai14 line, JAX stock 007914) to generate *Hic1*^*CreERT2/CreERT2 or* +^; *Rosa26*^*LSL−tdTomato/*+^ mice. To induce Cre-ERT2 nuclear translocation and reporter gene expression, ~ 8-week-old mice were injected intraperitoneally with 100 mg/kg of Tamoxifen (dissolved in sunflower oil) daily for 5 consecutive days, thereby generating *Hic1*^*CreERT2*^; *tdTom* reporter mice which were collected for analysis after a 10-day washout period.

Mice were housed under standard conditions (12 h light/dark cycle) and provided food and water ad libitum. For all experiments, litter mates of the same sex were randomly assigned to experimental groups. Animals were maintained and experimental protocols were conducted in accordance with approved and ethical treatment standards of the Animal Care Committee at the University of British Columbia.

### Fluorescence-activated cell sorting methodology

Fourth ventricle CP was collected from dissected brains of *Hic1*^*CreERT2*^*; tdTom* reporter mice using #5 forceps and transferred to a 1.5 mL Eppendorf tube with 500 μL DMEM until 13 CPs were dissected. CPs were then transferred into a 35 mm petri dish with 500 μL enzyme cocktail containing 1.5 U/mL Collagenase D (Roche 11 088 882 001) and 2.4 U/mL Dispase II (Roche 04 942 078 001), 2.5 mM calcium chloride and triturated regularly for 45 min using a P1000 pipette on a 37 ℃ hot plate. Following digestion, the cell suspension was transferred to a 1.5 mL Eppendorf tube on ice and quenched using 500 μL FACS buffer (2% FBS in 2 mM EDTA). The petri dish was then rinsed using two 250 μL washes to collect any remaining cells. The suspension was then centrifuged for 5 min at 500×*g* in a 4℃ refrigerated centrifuge. The supernatant was aspirated using a P1000 pipette and the cell pellet was resuspended in 500 μL antibody cocktail (FACS buffer with anti-CD45-Alx647, anti-CD31-Alx647 and anti-Ter119-Alx647) and left for 30 min to stain in the dark on ice. Stain was then washed using 1 mL FACS buffer and cells were pelleted at 4 ℃ for 5 min at 500×*g*. Supernatant was aspirated using P1000 pipette and cells were resuspended in 1 mL FACS buffer. The resulting cell suspension was filtered through a 35 μm filter into a 5 mL polypropylene FACS tube containing 2 μL 10 mg/mL Hoechst (33342, Invitrogen) and 1 μL propidium iodide (ThermoFisher #P1304MP, 1 μg/mL) to stain nuclei and diluted with an additional 3 mL FACS buffer (4 mL total). Tdtom^+^ Alx647^−^ cells were then sorted on an Influx (BD) using forward and side scatter along with Hoechst staining to distinguish live from dead cells and red blood, debris and doublets.

PGs were collected by first removing the calvaria and then the brain from *Hic1*^*CreERT2*^*; tdTom* reporter mice, taking care to ensure that the brain separates from the PG during removal. Eleven PGs were then removed from the sella turcica with forceps and carefully dissected into 5–6 pieces with fine scissors. PGs were then transferred into a 15 mL tube containing 3 mL of the enzyme cocktail described above and placed in an orbital shaker (Forma Scientific) at 37 ℃ rotating at 70 rpm, with tubes placed parallel to the ground to allow for optimal mixing. Samples were then incubated with a trituration step using a P1000 pipette every 15 min for 45 min. After the incubation, the enzyme cocktail was quenched by adding 12 mL FACS buffer and cellular material was extracted by centrifugation at 500×*g* for 5 min in a refrigerated centrifuge (Eppendorf 5810 R). Supernatant was removed with gentle suction and cells were resuspended in 800 μL antibody cocktail containing (FACS buffer with anti-CD45-Alx647, anti-CD31-Alx647 and anti-Ter119-Alx647) for 30 min, re-diluted in FACS buffer and centrifuged at 500×*g* for 5 min. Antibody-containing supernatant was removed and cells were re-suspended in 3 mL of FACS buffer containing 2 μL 10 mg/mL Hoechst (33342, Invitrogen) and 1 μL propidium iodide (ThermoFisher #P1304MP, 1 μg/mL). Tdtom^+^ Alx647^−^ cells were then sorted on an Influx (BD) using forward and side scatter along with Hoechst staining to distinguish live from dead cells and red blood, debris and doublets.

### Tissue fixation and cryosectioning

Tissue preparation, sectioning and imaging was performed as previously described [44]. Mice were transcardially perfusion fixed with 15 mL 10 mM EDTA in PBS followed by 15 mL 2% paraformaldehyde in PBS. Following perfusion, skin and excess connective tissue (skin, lower jaw and front teeth) were removed and sagittal slices of the entire skull were prepared at the midline using a sharp razorblade. Samples were then fixed in 2% paraformaldehyde for 48 h at 4 ℃, followed by three 24-h washes in decalcification solution (14% EDTA solution titrated to pH 7.5). Post-fixation, samples were washed in PBS then incubated in sucrose gradients of 10–20–30–40–50% (m/v) for 24 h each. Tissues were then immersed in OCT compound (Tissue Tek 4583) in plastic cryomolds (Polyscience 18646A) and frozen on dry ice, and stored at -80 ℃. Cryosections were cut (Thermo HM525 NX) at a thickness between 6 (for quantification) and 50 μm (for confocal imaging) and mounted on Superfrost Plus slides (VWR 48311-703).

For IF staining, slides were thawed on a 37° C slide warmer for 20 min and washed 3 times for 10 min in PBS to remove excess OCT compound. Next, autofluorescence was quenched for 45 min with 10 mg/mL sodium borohydride in PBS. Following borohydride treatment, slides were rinsed in PBS then permeabilized with 1 mL 0.1% Triton in PBS for 20 min. Slides were then washed with PBS and blocked for 90 min at room temperature with a solution containing 2.5% BSA (Sigma A7030) and 2.5% goat serum (Gemini 100–190). Following block, slides were stained overnight by incubation with primary antibodies typically diluted at indicated dilutions (Additional file 6: Table S1). The following day, primary antibodies were removed by 3 × 10 min washes in PBS then treated with Alexa Fluor 647- or 488- conjugated goat anti-rabbit or goat anti-rat secondary antibodies for 45 min at room temperature. After secondary incubation, slides were washed for 3 × 5 min washes with PBS and slides were counterstained with DAPI (ThermoFisher D3571, 600 nM) for 5 min, rinsed with PBS and mounted with Aqua Polymount (Polysciences 18,606).

### Image acquisition and quantification

**Fig. 1:**
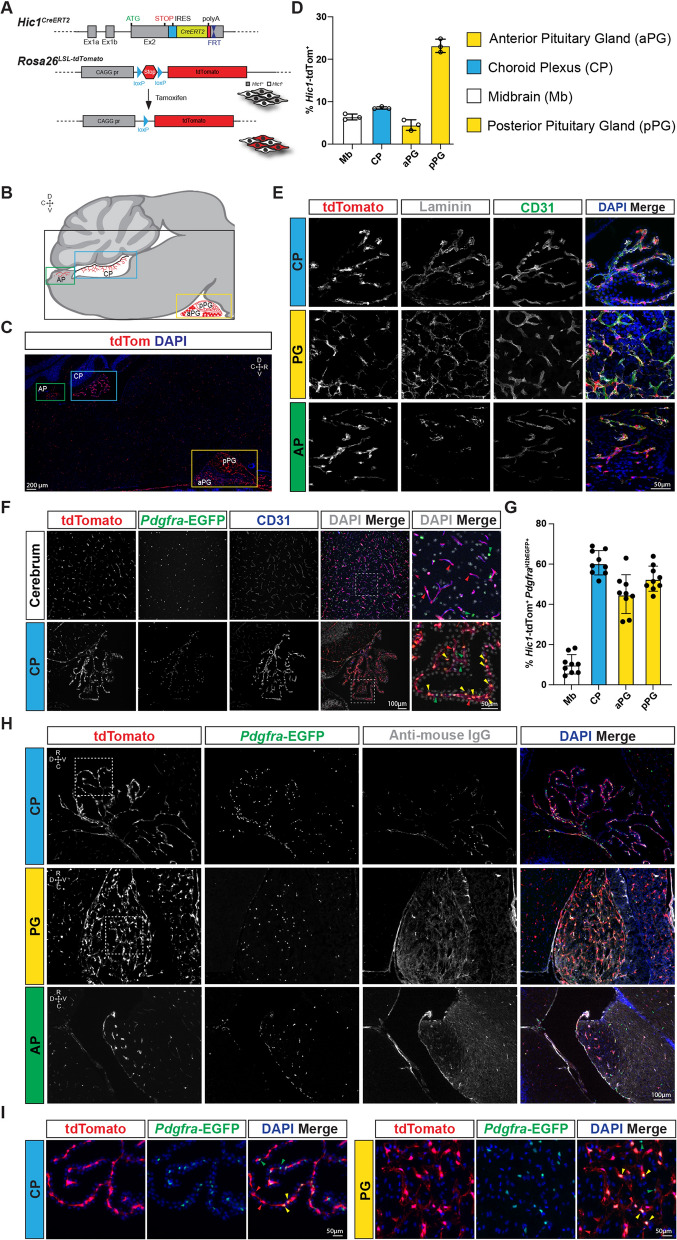
*Hic1* identifies mesenchymal cells in the CNS, including in the CP and PG. **A** Overview of the *Hic1*^*CreERT2*^*; Rosa26*^*LSL−tdTomato*^ labelling strategy. In this genetic background, tamoxifen administration leads to the indelible labeling of Hic1^+^ cells with tdTom. **B** Schematic cross-section of the mouse hindbrain, that highlights regions of abundant tdTom^+^ cells within the 4th ventricle CP and PG. Anatomical orientation is shown, D, dorsal; V, ventral; R, rostral; C, caudal. **C** Sagittal section of the hindbrain showing tdTom^+^ cells (red) within the 4th ventricle CP and PG. Samples were collected at 10 days post-TAM. **D** Enumeration of tdTom^+^ cells taken from sagittal sections of the midbrain (Mb), anterior PG (aPG), posterior PG (pPG) and 4th ventricle CP per total DAPI^+^ nuclei. **E** Representative sections of sagittal sections of the 4th ventricle CP and PG showing perivascular localization of tdTom^+^ cells in relation to the CD31^+^ endothelium and the laminin-containing extracellular matrix. **F** Representative sagittal sections of the 4th ventricle CP and cerebrum from *Hic1*^*CreERT2*^*; Rosa26*^*LSL−tdTomato*^*; Pdgfra*^*H2B−EGFP*^ mice. Red arrowheads represent singly tdTom^+^ cells, green arrowheads indicate singly EGFP^+^ cells and yellow arrowheads represent doubly positive cells. **G** Quantification of tdTom and GFP doubly positive cells in the indicated regions. **H** Co-localization of tdTom^+^ with GFP (*Pdgfra*^*H2B−EGFP*^) and endogenous IgG reveals an abundance of doubly positive cells in the permeable CP and PG. **I** High magnification insets from dotted squares shown in H for CP and PG. Red arrowheads represent singly tdTom^+^ cells, green arrowheads indicate singly EGFP^+^ cells and yellow arrowheads represent doubly positive cells

**Fig. 2:**
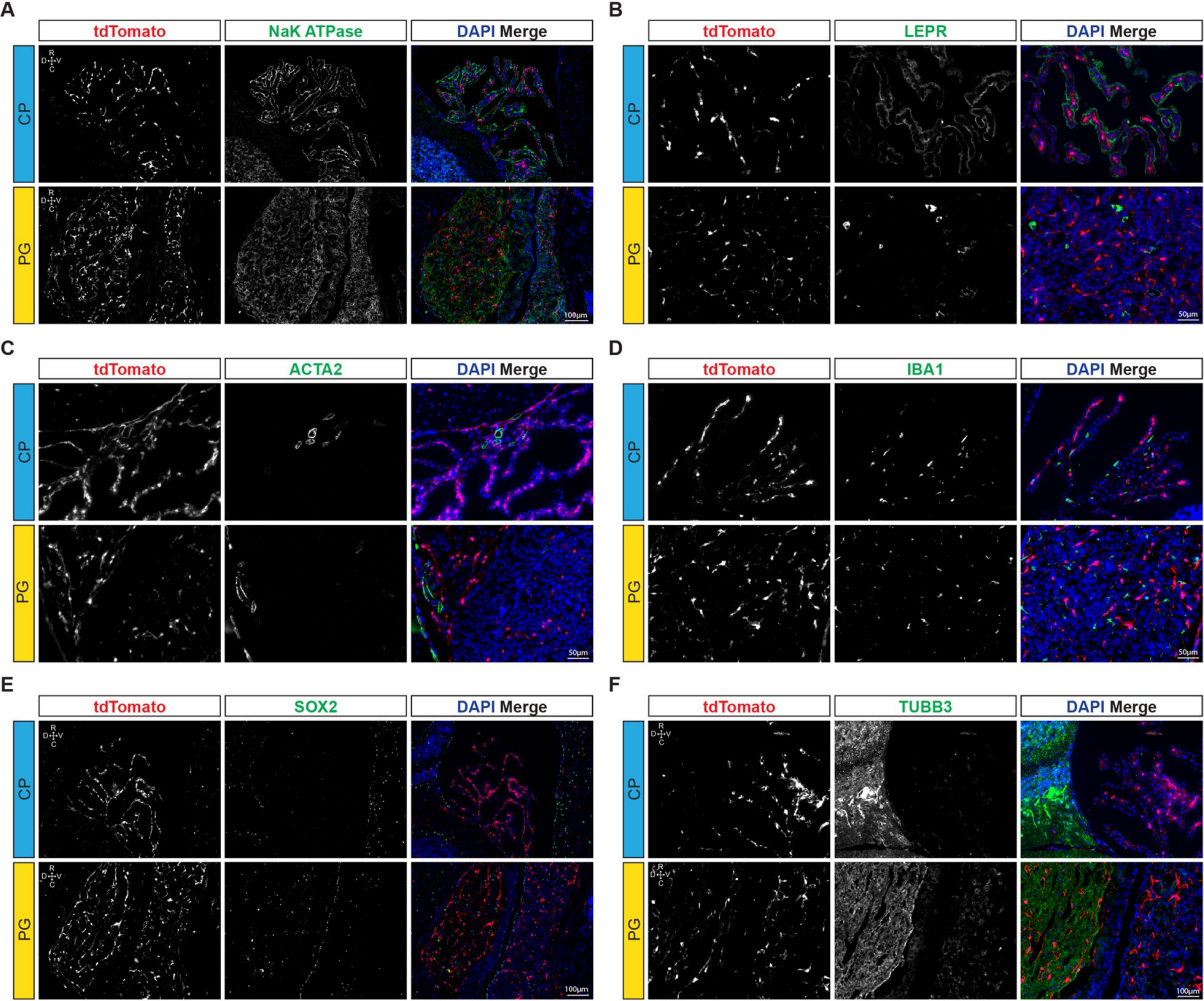
TdTom^+^ cells are restricted to mesenchymal cell types in the CNS. Representative sections counterstained with markers as indicated, tissue sections are in sagittal plane. **A** Na/K/ATPase staining of the 4th ventricle CP epithelium and PG endocrine cells. **B** LEPR staining of the CP epithelium and non-tdTom^+^ cells in the PG. **C** Staining with ACTA2 and identification of smooth muscle cells. **D** Microglial marker IBA1 occupies the same connective tissue compartment as tdTom^+^ cells in CP, but does not colocalize in 4th ventricle CP or PG. **E** SOX2, a marker of folliculostellate cells in PG and a putative neural stem cell marker, was totally absent from 4th ventricle CP, and does not co-localize with tdTom^+^ cells in the PG. **F** Pan-neural marker TUBB3 is absent from the 4th ventricle CP and does not co-localize with tdTom^+^ cells in the PG

Widefield epi-fluorescence microscopy (Figs. 1B, D, F, Fig. 2, Fig. 6, Additional file 2: Fig. S2A–C) was performed on an Olympus BX63 compound microscope. Confocal images (Fig. 1E, Fig. 5B and C, Additional file 2: Fig. S2A, B, D, E) were collected using a Nikon Eclipse Ti inverted microscope with a C2Si confocal system or a Nikon A1R HD25 system (Fig. 1F). Images LUTs were adjusted using Fiji software (ImageJ version 1.52a). Image quantification was performed by counting DAPI^+^ nuclei which were positive or negative for tdTomato and/or EGFP (3 images from 3 separate animals were used for quantification). For determination of the number of tdTom^+^ EGFP^+^ cells (Fig. 1G), tdTom^+^ cells were identified and examined for the presence of a DAPI^+^ nucleus along with positive nuclear EGFP signal (as above counts were performed on 3 images from 3 separate animals). Please note the scale bar in the bottom-right image applies for all images in the sub-panel, unless otherwise indicated. Images in all panels are oriented as shown in 1C (dorsal top, rostral right), unless otherwise indicated with an anatomical direction compass rosette.

### Alkaline phosphatase staining

Cryo-sectioned slides were washed 3 times with 1 mL tap water and treated with 1 mL alkaline phosphatase staining solution (0.1 mg/mL naphthol phosphate, 0.65 mg/mL Fast Red, 1 mg/mL MgCl_2_, and 40 μL dimethylformamide in 10% Tris solution) per slide and incubated for 60 min in the dark. Slides were then washed with tap water three times for 5 min, cover-slipped and imaged.

### Single cell RNA sequencing of choroid plexus

Thirteen CPs were processed in the same manner as for FACS sorting as described above. Target cells were enriched by FACS sorting into vacuum filtered collection media (DMEM containing 5% FBS) with propidium iodide (ThermoFisher #P1304MP, 1 μg/mL). Viable target cells were subsequently further purified, and debris reduced via a second sort. Cells were counted and quality control was determined by hemocytometer. Cells were then resuspended at a 3:1 TdTomato^+^: TdTomato^−^ ratio in 10 μL of collection media to obtain both the labelled population and representative other CP populations. The suspension was then input into a Chromium Controller (10X Genomics), captured and libraried with the Chromium single cell 3′ reagent kit v3 (10X Genomics). The cDNA libraries were sequenced on a Nextseq 500 (Illumina) to a minimum depth of 50,000 reads per cell. A transgenic reference genome was generated by the concatenation of the sequences for tdTomato to the mm10 reference genome and subsequent use of the cellranger *mkref* pipeline. Illumina sequencing output generated bcl files were de-multiplexed by bcl2fastq2. Alignment, demultiplexing, uniform manifold approximation and projecting (UMAP) and differential expression was performed using the cellranger *count* pipeline. Graphical outputs were then generated in the Loupe cell browser program v 5.0.0 (10X Genomics). The resulting UMAP was cleaned by filtering out cells under 5000 UMI (mostly low-quality fibroblasts) and removing doublets; identified by co-expression of established fibroblast markers (*Prrx1* and *Mfap4*) with established non-fibroblast markers (*Htr2c*, *Pecam-1*, *Ptprc* and *Cspg4*, for epithelium, endothelium, immune cells & pericytes respectively). Heat-maps were generated by the pheatmap package in R.

### Single cell RNA sequencing of pituitary gland

Eleven PGs were processed in the same manner as for FACS sorting as outlined above. Target cells were enriched by FACS sorting into vacuum filtered collection media (DMEM containing 5% FBS) with propidium iodide (ThermoFisher #P1304MP, 1 μg/mL). Viable target cells were subsequently further purified, and debris reduced via a second sort. Cells were counted and quality control was determined by hemocytometer. Cells were then resuspended at a 3:1 TdTomato^+^: TdTomato^−^ ratio in 42 μL of collection media to obtain both the labelled population and representative other PG populations. ScRNA-seq data was generated and processed in the same way as described for the CP.

### In silico single cell aggregation of mesenchymal cell populations

Following collection and independent analyses of CP and PG cell populations, cells from both tissues were aggregated using the cellranger *aggr* function. Fibroblasts and pericyte clusters were manually highlighted in Loupe Cell Browser v 5.0.0 (10X Genomics) based on the presence of *Hic1* expression and the resulting barcodes were reanalyzed using the cellranger *reanalyze* function*.* A heat-map from the resulting subpopulations was generated as described above.

### Data availability

All sequence data described in this paper have been deposited in GEO under the following accession numbers: GSE162733. Software used to analyze the data is either freely or commercially available.

### External data

An existing scRNA-seq dataset of the pineal gland [45] was referenced via GSE115723. Alignment, demultiplexing, uniform manifold approximation and projecting (UMAP) and differential expression was performed using the cellranger *count* pipeline. Graphical outputs were then generated in the Loupe cell browser program v 5.0.0. Images found in Additional file 4: Fig. S4 were sourced from the Allen Brain Atlas (Additional file 4: Fig. S4G, H; https://mouse.brain-map.org/gene/show/21503,) and the Allen Developing Brain Atlas (Additional file 4: Fig. S4 I, J; https://developingmouse.brain-map.org/gene/show/18184).

### Image quantification

Sample size determination was based on anticipated variability and effect size that was observed in the investigator’s lab for similar experiments. For image quantification, an n of 3 (3 animals with 3 sections per animal) was used. Representative images at 10X magnification were taken using the Olympus BX63 and total number of nuclei (labelled with DAPI) was enumerated along with total number of nuclei which were positive for tdTomato (red fluorescence in the absence of nuclei was not counted, Fig. 1D), followed by enumeration of total number of nuclei which were positive for both tdTomato and EGFP (Fig. 1G).

## Results

### The*** Hic1***^***CreERT2***^ reporter selectively labels mesenchymal cells within the 4th ventricle CP and PG

To identify the specific cell types marked by *Hic1*, brain sections from adult *Hic1*^*CreERT2/CreERT2*^; *Rosa26*^*LSL−tdTomato/*+^ reporter mice were analyzed following a 10-day washout period after TAM treatment (Fig. 1A). In other studies, we have found this regimen is effective at labelling Hic1^+^ mesenchymal cells [38–40]. Analysis of cryosections revealed robust expression of tdTomato (tdTom) in the CP and PG (Fig. 1B, C). Image-based quantification (via comparison of tdTom^+^ DAPI^+^ cells compared to tdTom^−^ DAPI^+^ nuclei) revealed that tdTom^+^ cells were more abundant in the CP and substantially more abundant in the posterior PG than they were in the adjacent brain. While the ratio of tdTom^+^ cells (to DAPI^+^ nuclei) in the anterior PG was lower than the rest of the brain, tdTom^+^ cells were nevertheless abundant, but were masked by the density of tdTom^−^ pituitary endocrine cells (Fig. 1D). Examination of the CP and PG at higher magnification revealed fluorescence restricted to perivascular cells located in close proximity to CD31^+^ vessels beneath a layer of extracellular laminin (Fig. 1E). Within the brain parenchyma, tdTom^+^ cells showed a similar morphology and were assumed to label pericytes and fibroblasts (for an in-depth examination of the populations of the brain vasculature, see [12]). To better characterize tdTom^+^ cells (which, in other tissues, can be divided into fibrogenic and pericytic subpopulations [38]) a *Pdgfra*^H2B−EGFP^ (*Pdgfra*^*H2B−EGFP/*+^) allele was introduced into the *Hic1*^*CreERT2*^*; tdTom* reporter background to distinguish Hic1^+^Pdgfra^+^ fibroblasts from Hic1^+^Pdgfra^−^ pericytes (Fig. 1F and G). In contrast to the whole brain, where fibroblasts were much less abundant than pericytes [12], in the CP and PG most tdTom^+^ cells were EGFP^+^ (Fig. 1F, G, and I). IgG, a blood component which does not pass the BBB or produce staining in the CNS was used to define areas with an intact BBB (Fig. 1H) [46]. As expected, the PG also showed a high level of staining for anti-mouse IgG (Fig. 1H). In aggregate, these findings indicate that *Hic1* identifies cells within the stroma of the CP and PG.

CP and PG were chosen as the focus for further analyses due to their accessibility and size relative to other smaller CVOs, and for their differential physiological secretory functions. Examination of immunostained cryosections of the CP and PG from *Hic1*^*CreERT2*^; *tdTom* reporter mice demonstrated that the tdTom signal was exclusive to mesenchymal cells, and did not co-localize with non- mesenchymal cell populations within the CP nor PG. Na/K ATPase, an established marker of CP epithelium [47], was detected at the apical side of the epithelium (Fig. 2A), as was LEPR (Fig. 2B). Within the PG, Na/K ATPase stained numerous cells both in the anterior PG and posterior PG, but was not appreciably detected in tdTom^+^ cells (Fig. 2A). LEPR was less abundant in the PG, and detected in only a small number of tdTom^−^ cells (Fig. 2B). In the CP, a population of tdTom^−^ smooth muscle cells was identified by α-SMA (ACTA2) expression. Adjacent to the PG, α-SMA was predominantly expressed in tdTom^−^ smooth muscle cells associated with large vessels, and low levels were also detected on PG resident perivascular tdTom^+^ cells (Fig. 2C). TdTom^+^ cells were also negative for the microglial marker IBA1 (Fig. 2D). SOX2, a marker of many neuronal lineages including PG glial cells [48], was expressed in a distinct region within the PG not associated with tdTom^+^ cells, and was not detected at all in the CP (Fig. 2E). Tubulin beta 3 (TUBB3) a neuronal marker, was abundant in both the glandular anterior PG and neural posterior PG, but was markedly absent from the CP (Fig. 2F). Across comparative sections, tdTom did not co-localize with neuronal derivatives, but was restricted to the perivascular compartment, suggesting that the tdTom^+^ cells represented brain pericytes or fibroblasts.

### *Hic1* identifies both pericytes and fibroblasts within the 4th ventricle CP and PG

**Fig. 3:**
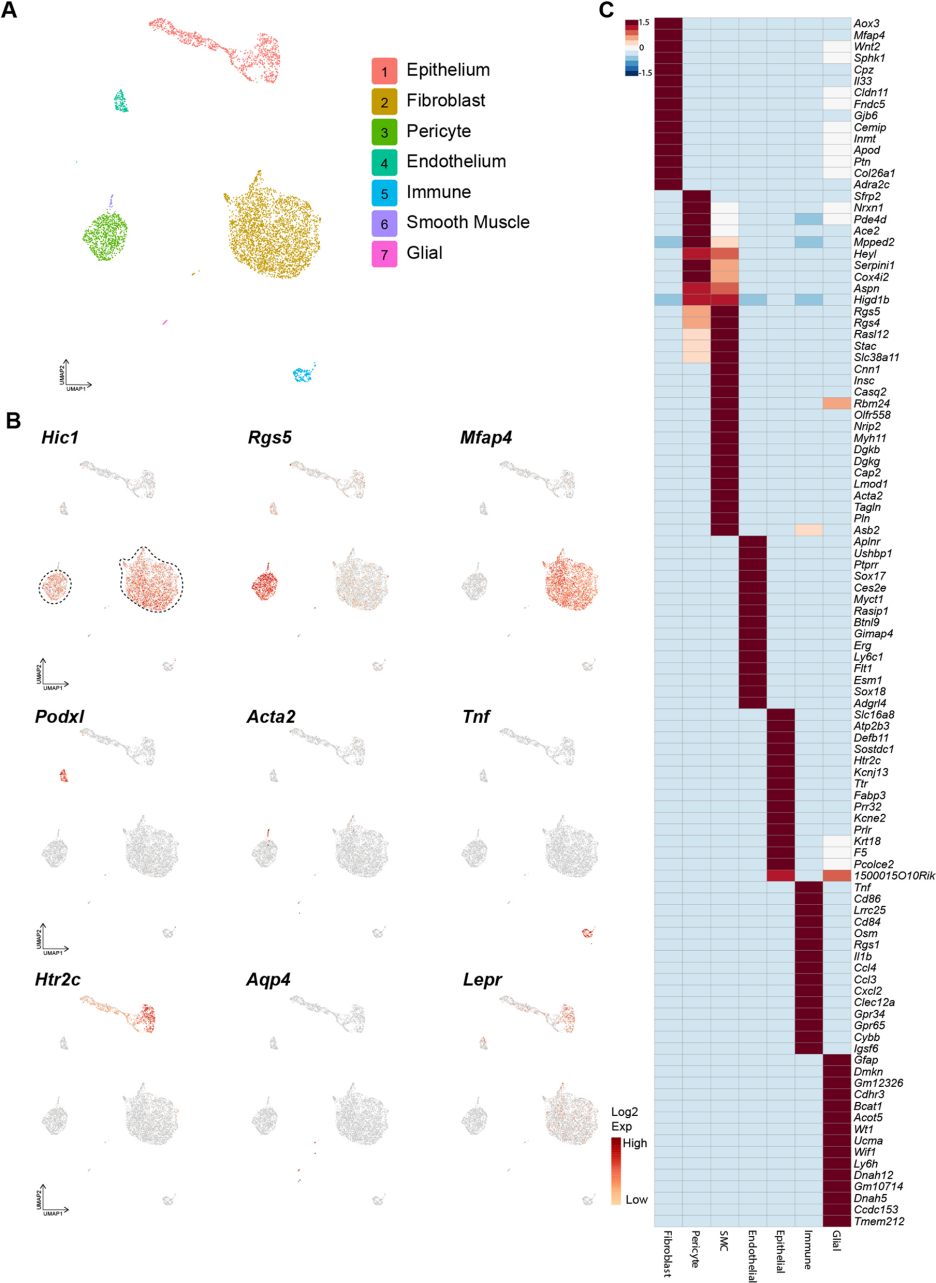
ScRNA-seq analysis of the fourth ventricle CP identifies various CP-resident cell types. **A** UMAP of scRNA analysis of CP, colored by cluster identity. **B** UMAP of genes marking mesenchymal cells (*Hic1*), pericytes (*Rgs5*), fibroblasts (*Mfap4*), endothelial cells (*Podxl*), smooth muscle cells (*Myocd*), immune cells (*Tnf*), CP epithelium (*Htr2c*) and glia (*Aqp4*). The dotted lines indicate which Hic1^+^ cells were included in the aggregate for reanalysis in Fig. 5. **C** Heat-map of enriched transcripts within the identified CP clusters

To determine the identity of the Hic1^+^ cells within the CP compartment, scRNA-seq was employed to characterize the transcriptome of CP-associated tdTom^+^ cells. A total of 5,791 cells were profiled from the 4th ventricle CP (3:1 ratio of tdTom^+^: tdTom^−^) and visualized using UMAPs (Fig. 3A). Distinct clusters were identified as CP fibroblasts (3,431 cells; *Mfap4*^+^*, Pdgfra*^+^*, Prrx2*^+^*, Wnt2*^+^), pericytes (878 cells; *Rgs5*^+^*, Cspg4*^+^*, Kcnj8*^+^) [49], CP epithelium (1,084 cells; *Ttr*^+^) [50], endothelium (163 cells; *Pecam1*^+^*, Sox17*^+^*, Sox18*^+^*, Flt1*^+^) [51–53], immune (163 cells; *Tnf*^+^) [54], smooth muscle cells (35 cells*; Acta2*^+^) [55], and glial-like cells (28 cells; *Aqp4*^+^*, Gfap*^+^) [56] (Fig. 3B and C). GFAP staining was not observed in the CP (Additional file 3: Fig. S3A), suggesting the glial-like cell population observed was possibly a product of contamination from brain parenchyma and not a distinct choroidal subpopulation. While *Acta2*^+^ smooth muscle cells exhibited a similar transcriptome to pericytes, they did not express *Hic1* and exclusively expressed canonical smooth muscle markers including *Tagln*, *Myh11, Myocd* and *Pln* (Fig. 3C) [43]. Consistent with previous studies in skeletal muscle [38], heart [39] and skin [40], expression of *Hic1* in CP scRNA-seq was restricted to pericyte and fibroblast clusters and was not observed in other cell types (Fig. 3B).

In contrast to the specificity with which *Hic1* labels mesenchymal cells, other common markers used in fibroblast or pericyte identification in other tissues showed either overlap between the two cell types, low labeling efficiency, or both, in the scRNA-seq dataset. *Lepr*, in addition to marking large populations of hippocampal and arcuate neurons [6] was restricted almost entirely to the epithelium in the CP (Fig. 2B). Consistent with a previous report [57], *Nes* was expressed, albeit stochastically, across multiple cell types in the CP. Of all the common markers examined, *Prrx1* (Additional file 3: Fig. S3B) showed the greatest overlap with mesenchymal cells, though it also labeled smooth muscle cells and some epithelial cells (and it should be noted that *Prrx1* is also abundantly expressed in astrocytes [8]). While PDGFRA can be found in a variety of cell types in the adult brain including oligodendrocytes and astrocytes [7], in combination with *Hic1*^*CreERT2*^; *tdTom* reporter mice, the inclusion of the *Pdgfra*^*H2B−EGFP*^ allele was useful to enable effective separation of fibroblasts (*Pdgfra*^+^) from pericytes, which expressed a transcriptome similar to that seen in pericytes in other tissues of the body including markers such as *Cspg4* and *Rgs5* (Fig. 3B and Additional file 3: Fig. S3B).

**Fig. 4:**
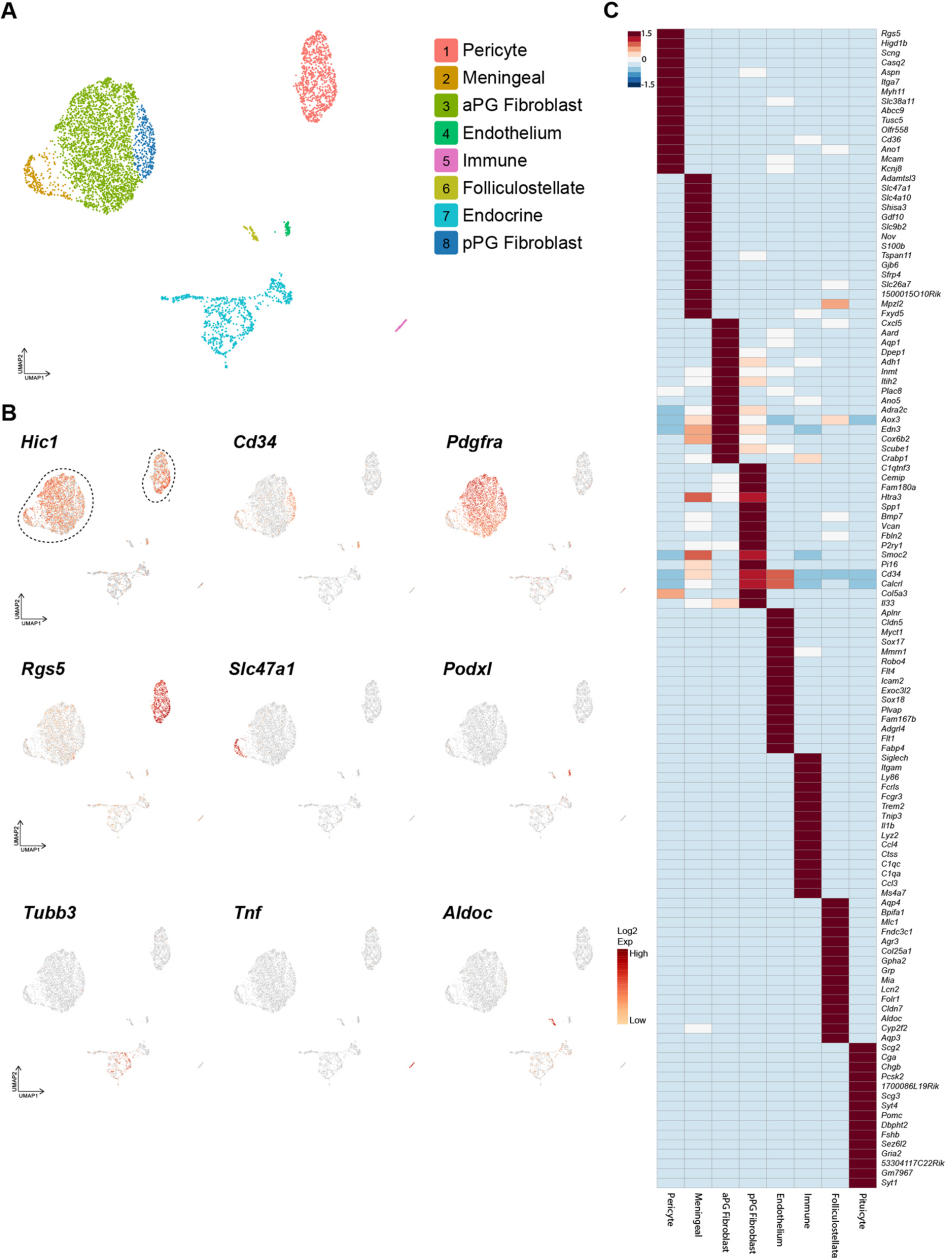
ScRNA-seq of tdTom^+^ cells isolated from the PG. **A** UMAP of scRNA analysis of PG, colored by cluster identity. **B** UMAPs of genes marking mesenchymal cells (*Hic1*), posterior PG fibroblasts (*Cd34*), anterior pituitary (aPG) fibroblasts (*Pdgfra*) pericyte (*Rgs5*), meningeal cells (*Slc47a1*), endothelium (*Podxl*), pituitary endocrine cells (*Tubb3*), immune (*Ptprc*) and glial cell (*Aldoc*) populations. The dotted lines indicate which Hic1^+^ cells were included in the aggregate for reanalysis in Fig. 5. **C** Heat-map of transcripts within the identified PG clusters

The PG scRNA-seq dataset (Fig. 4A) contained 5,232 cells and generated clusters within a UMAP of *Hic1*^+^ mesenchymal cells including pericytes (901 cells*; Rgs5* ^+^) [49], meningeal fibroblasts (264 cells; *Slc47a1*^+^) [58], anterior PG fibroblasts (3,040 cells; *Pdgfra*^+^*, Pax9*^+^*, Aqp1*^+^*, Cldn11*^+^), and posterior PG fibroblasts (315 cells; *Cd34*^+^*, Bmp7*^+^*, Spp1*^+^*, Pi16*^+^). *Hic1*^−^ cells included pituitary endocrine cells (809 cells; *Pomc*^+^*, Fshb*^+^*, Prl*^+^) [24], folliculostellate cells (79 cells; *Aldh1a2*^+^*, Sox2*^+^) [59], endothelium (67 cells; *Podxl*^+^) [53] and immune cells (62 cells; *Tnf* ^+^) [54] (Fig. 4B and C). Unlike the CP, a distinct smooth muscle cells cluster was not evident, and expression of genes associated with smooth muscle function, such as *Acta2* and *Tagln* were present at a low level across the entire pericyte cluster, whereas smooth muscle cell genes such as *Cnn1* were not detected.

**Fig. 5:**
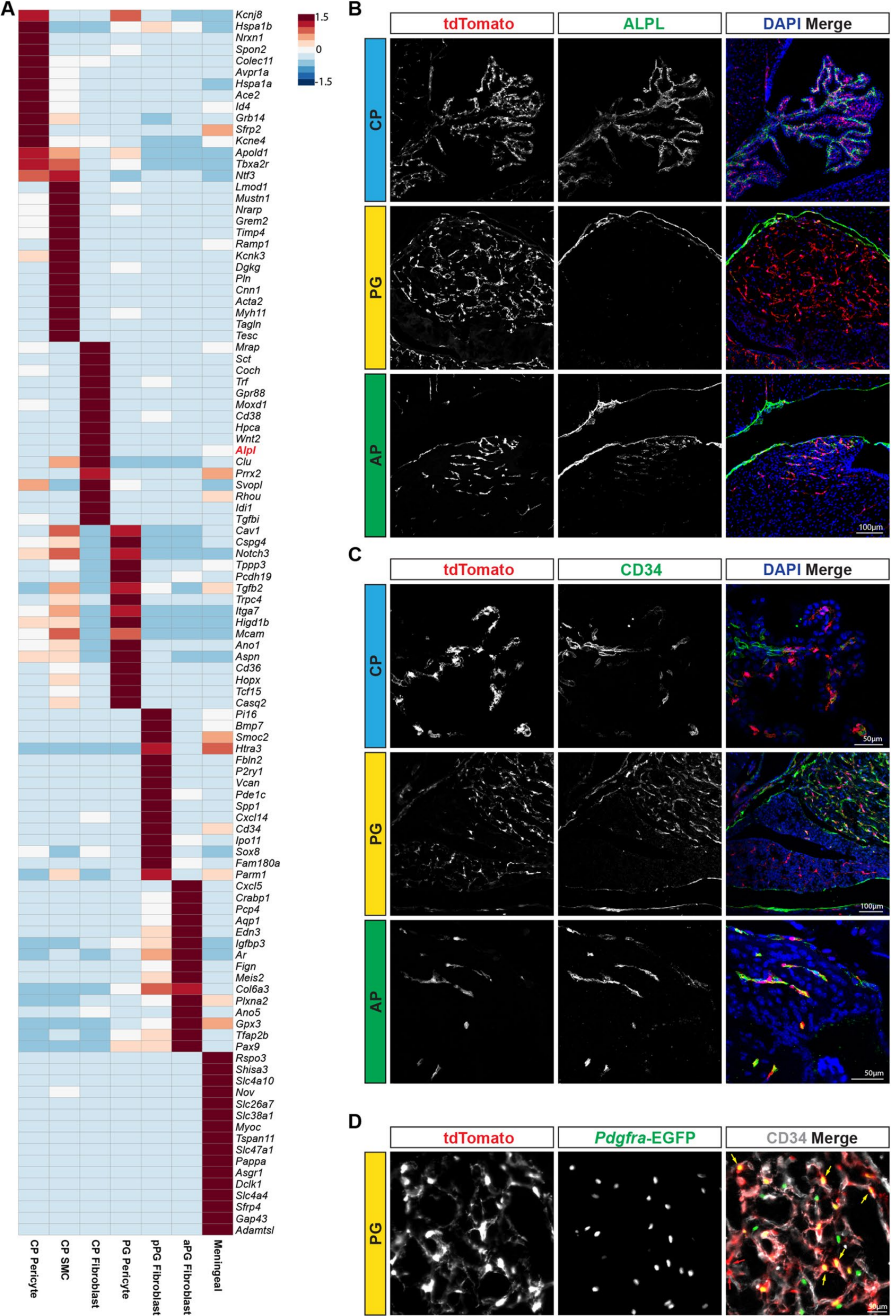
Analysis of differentially-expressed genes in 4th ventricle CP and PG. **A** Heat-map of differentially expressed genes within indicated Hic1^+^ subsets. **B** ALPL can be identified throughout the mesenchymal compartment of the 4th ventricle CP (top), but only in the outer meningeal layer of the PG (middle). **C** CD34 is predominantly localized to endothelial cells within the 4th ventricle CP (top panel). CD34 is strongly expressed within the posterior PG, and is weakly expressed in adjacent intermediate PG or anterior PG. **D** Within the PG, CD34 is localized to the tdTom; EGFP doubly positive cells (yellow arrows), but not the singly positive tdTom cells (red arrows). CD34 is also present on endothelial cells

Within the identified cluster of pituitary endocrine cells (Fig. 4A and C), multiple subpopulations of PG endocrine cells could be identified (Additional file 1: Fig. S1). Within the generated endocrine cell-specific heat-map, there were identifiable clusters of corticotrophs (129 cells, *Tbx19*^+^*, Rspo3*^+^*, Tnnt1*^+^*, Pomc*^+^), gonadotrophs (27 cells, *Fshb*^+^*, Gnrhr*^+^*, Lhb*^+^, *Hpgd*^+^), somatotrophs (388 cells, *Gh*^+^*, Rxrg*^+^*, Wnt10a*^+^*, Pappa2*^+^) lactotrophs (125 cells, *Prl*^+^*, A2m*^+^*, Ramp1*^+^*, Spock1*^+^) and melanotrophs (19 cells, *Pax7*^+^*, Prrx1*^+^*, Cd24a*^+^*, Oacyl*^+^).

### Comparative analysis of mesenchymal cells within the CP and PG

Given the similarities between CP and PG mesenchymal cells, scRNA-seq data from the major Hic1^+^ clusters were aggregated to identify potentially unique features between these two tissue compartments (Fig. 5A). While expression of many canonical fibroblast genes, such as *Pdgfra, Lum* and *Col1a1* were shared across fibroblast clusters, region-specific genes were identified for anterior PG (*Aqp1*, *Cxcl5*) posterior PG (*Cd34*, *Pi16*) and CP (*Alpl, Gpr88*) (Fig. 5A). Meningeal cells sequenced from the PG expressed *Hic1* and *Alpl* along with some mesenchymal cell markers such as *Col1a1* and *Pdgfra*, but showed little to no expression of markers associated with pericytes (such as *Cspg4*, *Rgs5*, *Kcnj8*) and expressed a host of markers not detected in other compartments, including *Slc4a10*, *Tspan11* and *Slc47a1*.

Within the large cluster of *Pdgfra*^+^ fibroblasts in the PG (Fig. 4), two sub-clusters of cells could be distinguished based on gene expression. The major sub-cluster contained ~ 2700 cells that expressed *Cxcl5*, *Aqp1* and *Crabp1* (Fig. 4C and Additional file 3: Fig. S3D). The second cluster, while closely associated in UMAP space, can be identified by specific expression of *Cd34* (Fig. 4B and Additional file 3: S3B), *Pi16*, and *Bmp7*, as well as the absence of *Cldn11*, which is shared between both the major sub-cluster and the meningeal cluster (Fig. 4C, Additional file 3: Fig. S3C and D). Immunofluorescence of CD34 detected strong expression in the posterior PG and not in the anterior PG (Fig. 5C), suggesting that this population is concentrated in the fibroblasts of the posterior PG. Furthermore, consistent with the scRNA-seq data, CD34 is found on the tdTom^+^ EGFP^+^ cells within the PG and not the tdTom singly positive cells (Fig. 5D). In the CP, CD34^+^ cells could be identified but did not co-localize with tdTom^+^ cells, and likely represented endothelial cells (which also express CD34) (Fig. 5C).

Alkaline phosphatase (*Alpl*) was found to be distinctly expressed in CP fibroblasts in comparison to PG (Fig. 5A). While the CP is known to express *Alpl* [60], this study further supports that CP associated fibroblasts express *Alpl*. In contrast, within the PG, the majority of cells (as by scRNA-seq) did not express *Alpl* (Fig. 5A), with the exception of the presumptive meningeal sub-cluster (Fig. 4C). Immunofluorescence detection of ALPL (Fig. 5B), coupled with secondary validation through chromogenic alkaline phosphatase staining (Additional file 2: Fig. S2C), demonstrated that ALPL is abundant in the CP stroma. Within the PG, ALPL staining was absent, but could be observed in the meningeal cells surrounding the PG (Fig. 5B). Further analysis of the outer PG revealed tdTom^+^ cells embedded in an ALPL-rich matrix, which forms at the PG-brain interface including at the diaphragma sellae of the PG (Additional file 2: Fig. S2A, B).

### Mesenchymal cells express genes relevant to specific vascular properties

**Fig. 6:**
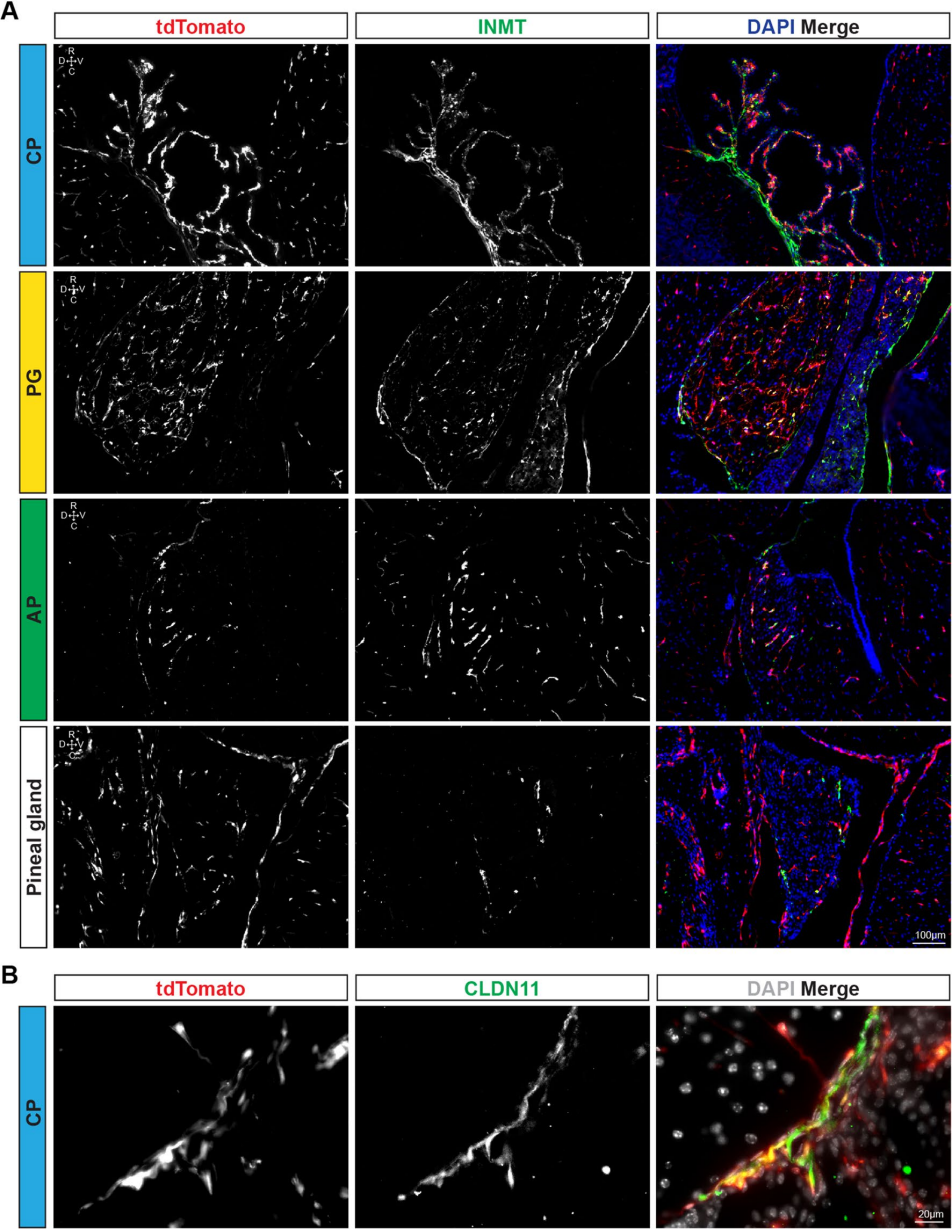
Fibroblasts are the exclusive producers of INMT in the brain. **A** INMT co-localizes with tdTom^+^ cells in the 4th ventricle CP**,** PG, AP and pineal gland (sagittal sections), but not with pinealocytes. **B** CLDN11 is also primarily expressed by tdTom^+^ cells in the 4th ventricle CP

We further validated the expression of proteins encoded by transcripts identified within the CP mesenchymal compartment using histological analysis. In particular, indole-n-methyltransferase (*Inmt/*INMT) was found to be specifically expressed in fibroblasts in the CP and PG—and not within pituitary endocrine cells or CP epithelium (Additional file 3: Fig. S3C). Given that this finding is in apparent contradiction to recent literature about *Inmt*, which reported detection of *Inmt* transcripts within the CP epithelial cells, and especially within pinealocytes [61], we further validated our finding using an INMT specific antibody (Fig. 6A). Congruent with the scRNA-seq analysis, INMT co-localized with tdTom^+^ cells extensively in these areas and it was absent from the CP epithelial layer (Fig. 6A). Similarly, in the PG, there was extensive colocalization between INMT immunostaining and Hic1-tdTom^+^ cells (Fig. 6A). The AP, which possessed an abundance of *Hic1*^+^*/Pdgfra*^+^ cells, also contained INMT^+^ cells (Fig. 6A), whereas the pineal gland (where much of the published literature on INMT is focused) was INMT^−^, outside of occasional (tdTom^+^) leptomeningeal cells on the periphery of the gland (Fig. 6A). To further validate our inability to detect INMT inside the pineal gland proper, we referenced a publicly available scRNA-seq data set ([45], GSE#115723, Additional file 4: Fig. S4A–F) taken from rat pineal gland. While being mostly composed of (*Inmt*^*−*^) pinealocytes, it also contained a population of resident fibroblasts termed Vascular Lepto-Meningeal Cells (VLMC). *Inmt,* as well as *Hic1* and *Cldn11* transcripts were detected in the VLMCs, but not in *Ddc*^+^ pinealocytes. Expression of *Inmt* in the adult brain is thus restricted to mesenchymal cell types.

Claudin-11 (CLDN11, *Cldn11*) is a cell adhesion molecule known to contribute to oligodendrocyte tight junctions which have been found in the CP [62], and has since been assumed to similarly contribute to CP tight junction formation in CP epithelium. However, *Cldn11* was found to be expressed exclusively in fibroblasts within the CP (and not in epithelial cells) (Additional file 3: Fig. S3C). While *Cldn11* transcripts were detected in scRNA-seq across CP, anterior PG and meningeal fibroblast clusters, appreciable CLDN11 immunofluorescence signal was observed exclusively in the CP (Additional file 3: Fig. S3C and Fig. 6B). In addition, expression of CLDN11 overlapped with tdTom^+^ cells in the CP (Fig. 6B). Furthermore, in situ hybridization of embryonic brains (Allen Developing Brain Atlas, embryonic day 13.5 and 15.5; Additional file 4: Fig. S4I and J) at timepoints before myelination but after CP formation revealed *Cldn11* transcripts within the CP stroma as well as the developing meningeal layer around the brain. Consistent with these observations, a recent study employing a *Cldn11-*CreERT2:Ai14 tdTomato reporter mouse strain [63], showed expression of *Cldn11* in both choroid plexus fibroblasts and arachnoid barrier cells.

Several transcripts that encode various proteins associated with either CP function or identity, but have not been attributed to a particular cell type (*Cldn11*[62] *Wnt5a* [64], *Tcf21* [65], *Avpr1a* [66], *Nrxn1* [67], *Fndc5* [68] and *Gjb6* [69]), exhibited robust expression specific to Hic1^+^ mesenchymal cells (Additional file 3: Fig. S3C).

Recent studies of the other circumventricular organs, in particular the organ vasculosum of the lamina terminalis (OVLT) and the subfornical organ (SFO), have also recently employed scRNA-seq to provide insights into the transcriptomic patterns in permeable brain regions [70]. However, beyond mentioning that “fibroblast” and “pericyte” populations were identified, these populations were not considered further. Herein, this dataset was reanalyzed to further explore the identity and features of brain-associated mesenchyme described herein (Additional file 5: Fig. S5A and B). It is worth noting that the cell preparation used by Pool et al. [70] was intended to capture neuronal populations and not vascular ones, and based on similarities to a reported type I fibroblast [12], the captured fibroblast populations may originate from surrounding meninges. Nevertheless, our finding that *Hic1*, *Inmt* and *Postn* were robustly expressed in their fibroblast cluster (Additional file 5: Fig. S5C and D), is consistent with our captured mesenchymal populations. The putative receptor for the hypothesized *Inmt*-produced methyl-tryptamines, *Htr2c*, could also be detected in excitatory neuron populations in both the OVLT and SFO (Additional file 5: Fig. S5C and D). We further analyzed the OVLT via immunofluorescence, where a distinct subpopulation of tdTom^+^, EGFP^+^, INMT^+^ cells could be found within a vascular plexus (Additional file 5: Fig. S5E). However, in contrast to the fibroblasts of the posterior PG or CP, neither *Cd34/CD34 n*or *Alpl/ALPL* were detected in the OVLT fibroblasts by antibody staining, phosphatase activity or in the available scRNA-seq data, suggesting that these may be represent specific markers for fibroblasts within PG and CP/meninges, respectively.

## Discussion

### ***Hic1***^***CreERT2***^ selectively labels brain-associated mesenchymal populations

Our study demonstrates that the *Hic1*^*CreERT2*^*; tdTom* mouse line specifically identifies mesenchymal populations consisting of pericytes and fibroblasts or their derivatives, without labelling other cell types within the brain. TdTom^+^ cells were found throughout the brain, decorating blood vessels in a manner commonly associated with mesenchymal cells using other markers such as *Cspg4* [49] and *Pdgfra* [12]. TdTom reporter expression was specific to mesenchymal cells, and did not co-localize with markers for endothelium, smooth muscle cells, micro- and macro- glial cells or neurons. Thus, it appears that *Hic1* expression is restricted to mesenchymal cells within the brain. In the CP and PG, comparable ‘fibroblast’ and ‘pericyte’ subpopulations consistently emerged, congruent with work using *Hic1*^*CreERT2*^ in other tissues [38, 39]. To complement recent single-cell studies of CP [22, 71, 72] and PG [24, 25], we analyzed distinct subgroups of newly characterized mesenchymal cells at further resolution.

### Fourth ventricle choroid plexus fibroblasts

Fourth ventricle CP fibroblasts expressed novel genes which were not observed in other cell types, including but not limited to *Mfap4* and *Il33*. CP fibroblasts shared multiple markers with parenchymal brain fibroblasts consistent with those identified by Vanlandewijck et al. [12], such as *Col1a1, Lum* and *Mmp2*, they did not express others such as *Cdh5* and *Slc6a13*, and expressed an array of distinct genes including *Gpr88, Coch* and *Tgfbi*. It would appear, given these comparisons, that the identified CP fibroblasts correspond to “Type II fibroblasts” which are briefly mentioned in Vanlandewijck et al. [12]. Many genes identified by Vanlandewijck et al. [12] as distinct to brain fibroblasts, including *Col26a1, Moxd1,* and *Cemip* are in fact specific to Type II (CP) fibroblasts. Some genes which were largely specific to Type II fibroblasts in the brain, such as *Alpl* and *Tcf21*, were found to be expressed in lung fibroblasts but not in other brain MP populations [12].

### Posterior pituitary fibroblasts

Fibroblasts from the posterior PG contained a remarkable proportion of a distinct fibroblast subpopulation (Fig. 1D), which has hitherto remained largely uncharacterized (PG fibroblasts are usually referred to in the collective, if they are mentioned at all) [24–26]. Only a small number of genes distinguished the anterior and posterior PG fibroblast subpopulations, and most fibroblast-associated genes (*Hic1, Prrx1, Pdgfra, Des*) are expressed at comparable levels across anterior and posterior PG fibroblast clusters (Fig. 4B, Additional file 3: Fig. S3B and S3C). Interestingly, although all three regions exhibit greater vascular permeability than the rest of the brain, the CP and anterior PG exhibit limited innervation, while the posterior PG is highly innervated. Beyond their morphological differences, posterior PG fibroblasts can be identified through their abundant expression of *Cd34* (Additional file 2: Fig. S2D). Along with *Cd34*, genes found in posterior PG fibroblasts (*Pi16* [73], *Spp1* [74] and *Vcan* [75]), are known to contribute to increased vascular permeability in the brain (Fig. 4C). In summary, the distinct subpopulation of fibroblasts which was uncovered in the posterior PG express a number of permeability-associated markers which may prove a valuable starting point for future studies of the regulation of blood–brain barrier function in more innervated regions.

### Anterior pituitary fibroblasts

Fibroblasts from the glandular anterior PG constituted the bulk of the PG fibroblasts within the PG dataset (Fig. 4A). This seems to be a result of the anterior PG being both larger and more cell-dense than the posterior PG (the posterior PG seems to have substantially more fibroblasts as a portion of total cells present, but far fewer cells overall). Anterior PG fibroblasts were identified *in-situ* as Hic1-tdTom^+^ CD34^−^ cells, and the Cd34^−^ cluster identified in our scRNA-seq data demonstrated the expression of distinct positive markers, including *Aqp1*, *Cxcl5* and *Crabp1* (Additional file 3: Fig. S3D). Congruent with existing literature, scRNA-seq data found that mesenchymal cells were the primary producers of *Col1a1* and *Col3a1* [27], and anterior PG fibroblasts also expressed mesenchyme-associated transcripts *Prrx1* and *Prrx2* [28]. While *Nes* appeared to be expressed in some anterior PG endocrine cells, it was more consistently expressed in pericytes, suggesting that the *Nes*^+^ cells identified in PG [76] probably represent pericytes (and not fibroblasts) in this region.

### Pituitary & CP pericytes

In the PG, like the rest of the brain [12], *Acta2* was detected within a subset of pericytes, whereas CP pericytes exhibited negligible expression. *Ace2*, the gene encoding the suspected cellular entry target for COVID-19, was found to be enriched in CP pericytes (Additional file 3: Fig. S3C), with lower levels observed in PG pericytes and CP fibroblasts (and on BBB pericytes [12]). This finding was particularly noteworthy in light of recent findings that the CP is especially susceptible to COVID-19 [77], and that pericytes specifically within the CP are an established medium of invasion for the Zika virus [78].

### Meningeal fibroblasts

Within the PG scRNA-seq dataset, a cluster of *Hic1*^+^ *Slc47a1*^+^ cells with a distinct pattern of gene expression (Fig. 4B), corresponded to a meningeal subpopulation previously described as arachnoid barrier cells based on expression of the transporter *Slc47a1*, as well as *Abcb1* and *Abcg2* [79]. Both *Abcb* transcripts encode transport proteins typically associated with the arachnoid barrier. However, characterization of embryonic meningeal cells has revealed a similar (*Slc47a1*^+^*, Abcb1*^+^*, Abcg2*^+^*, Alpl*^+^*)* population which was reported to represent dural resident cells based on the expression of *Alpl* and *Foxc2* [58]. While the meningeal cluster identified herein shares a number of genes with the reported ‘embryonic dura’ population [58], it is worth noting that some genes (*Mgp, Crabp2*) were not detected in our meningeal cluster and therefore may be embryo specific. Early developmental studies indicated that the PG is surrounded specifically by dural cells, while the leptomeningeal mesenchyme (including cells, which in the brain proper, would form the arachnoid barrier as well as the pia mater) ultimately forms the stroma of the PG [80, 81]. These observations, coupled with the analysis of Hic1-tdTomato ALPL co-localization, suggests that *Hic1*^+^ *Slc47a1*^+^ cells represent dural barrier cells which contribute to the outermost meninges of the brain, corresponding to a similar subpopulation of mesenchymal cells identified earlier in the spinal dura [42].

### Other circumventricular organs: AP, OVLT and SFO fibroblasts

While the small size of the circumventricular organs and limited number of fibroblasts confounds their analysis through single-cell RNA sequencing (at least in mice, where large numbers of animals would be needed to adequately enrich for and capture rarer populations, such as fibroblasts), histological analysis and comparison to published findings has enabled further query of the fibroblasts in CVOs. These regions have been previously identified as containing an abundance of *Cspg4*^+^, *Pdgfrb*^+^ pericytes [82]. Based on our analysis of the scRNA-seq data collected from the CVOs by Pool et al. [70], it seems that fibroblasts within the CVOs more closely resemble fibroblasts in the rest of the brain in comparison to fibroblasts within the CP or PG (e.g. they do not express CP fibroblast markers such as *Gpr88* or *Alpl*, nor do they express posterior PG-specific markers such as *Cd34*).

*Inmt* is expressed in CP (‘type II’), as well as at lower levels in brain resident (‘type I’), fibroblasts [12]. This is further evidenced by a general paucity of INMT immunofluorescence staining in gray and white matter, in contrast with the brightly labelled permeable regions (including the PG), and this trend was maintained in the OVLT (Additional file 5: Fig. S5E). Furthermore, as observed in the CP (Fig. 3C) and in neuronal projections into the posterior PG [83], receptors sensitive to INMT-synthesized methyltryptamine products, including those encoded by *Htr2c* and *Htr7* were abundantly expressed in nearby secretory cells (in this case, neurons) in the OVLT and SFO.

### Novel markers of neurovascular mesenchyme in permeable vasculature

Mesenchymal cells play a fundamental role in maintaining CP structural integrity, enzymatic metabolism, and secretion of trophic factors. Labelling using the Hic1-tdTom fluorescent reporter revealed the efficacy and specificity of *Hic1*^*CreERT2*^ in identifying neural tissue resident mesenchymal cells, and allowed for their quantification. Once the presence and scope of CP mesenchyme was established, scRNA-seq was performed to analyze PG cells at cellular resolution, which were then validated using immunofluorescence. This study provides insight into novel contributions of mesenchymal cells in the CP and PG to brain function, including the exclusive expression of *Inmt, Cldn11* and *Wnt5a* (previously attributed to epithelial lineages) and has further resolved distinct subpopulations of mesenchymal cells within the anterior and posterior PG.

In the adult brain, ALPL metabolizes pyridoxal-phosphate (B6) to pyridoxal, the active cofactor required for the synthesis of inhibitory neurotransmitter gamma-aminobutyric acid [84]. While several scRNA-seq datasets which profile brain endothelial cells [12, 85] suggest they are a source of ALPL in the brain, the CP is also known to play a substantial role in the accumulation and release of both pyridoxal and pyridoxal-phosphate [86]. Relative to mesenchymal cells in the PG parenchyma, meningeal cells strongly expressed ALPL, where the gene has an established role regulating both meningeal and calvaria development [87]. Further studies are required to reveal the specific contributions of endothelial vs mesenchymal ALPL in their respective zones of the blood–brain barrier, CP and meninges.

Indole-n-methyl transferase (*Inmt*/INMT) is an enzyme found throughout the body, primarily in the lungs [88]. It is best known for its capacity to methylate single amino acid derived compounds including tryptamine, serotonin and phenethylamine [89]. While the function of INMT remains controversial [90], a recent study suggested that INMT was expressed in CP epithelium, as well as the pineal gland, where it was proposed to synthesize the psychoactive compound N,N-dimethyltryptamine [61]. However, based on immunofluorescence and scRNA-seq analysis, in regions associated with the CNS, *Inmt*/INMT is restricted to fibroblasts (Fig. 6A, Additional file 3: Fig. SS3C). This finding is corroborated by scRNA-seq studies from other groups (Additional file 4: Fig. S4A–F) [70]. Interrogation of the online tool associated with a whole brain vasculature scRNA-seq dataset [12], revealed abundant *Inmt* expression in brain fibroblasts (particularly enriched in ‘type 2 fibroblasts’, which express similar markers to CP fibroblasts including, *Tcf21, Wnt2* and *Gpr88* and likely constitute the same subpopulation)*.* Furthermore, in situ hybridization studies from the Allen Brain Atlas show *Tcf21,* as well as *Inmt*, restricted to the CP (Additional file 4: Fig. S4G and H). In a recently published pineal gland scRNA-seq dataset, *Inmt* was almost exclusively expressed in Hic1^+^ fibroblasts (Additional file 4: Fig. S4F), which represent the vascular lepto-meningeal cells, and was absent within *Ddc*^+^ pinealocytes [45] (Additional file 4: Fig. S4C). The INMT substrate serotonin is known to accumulate within the CP [91], possibly because of the plasma membrane monoamine transporter (PMAT) encoded by *Slc29a4* which is expressed in CP epithelium [92, 93]. Furthermore, given the high expression levels of the serotonin receptor HTR2C in CP epithelium, which is particularly sensitive to methyltryptamines [94], the lack of investigation into the role of INMT in the CP is notable. It remains to be determined if INMT is required or sufficient for methyltryptamine production in vivo—a recent study showed that, at least in rat, INMT is neither necessary nor sufficient for the production of endogenous DMT [95]. In contrast, HTR2C has been studied extensively and functional associations have been found between receptor activation and important CP functions, such as promoting insulin production from CP epithelium [96] and cerebrospinal fluid exocytosis [97]. Herein, we demonstrate that expression of *Inmt* in the brain is restricted to fibroblasts, and is not detected in CP epithelium, PG glandular cells or pinealocytes.

CD34 is a membrane phosphoglycoprotein in the sialomucin ‘CD34-family’, which also includes podocalyxin and endoglycan [98]. While CD34 is best known for its clinical use as a marker for hematopoietic stem cells in bone marrow, it has also been found on a variety of cell types including, muscle satellite cells [99], endothelial cells ([100], Additional file 3: Fig. S3B) and some other subpopulations of mesenchymal cells within the bone marrow [101, 102]. In the brain, increased expression of CD34 is associated with increased vascular permeability in epileptic neovascularization [103] and brain injury [104]. However, since neurological insults are often also associated with endothelial proliferation, further studies are required to distinguish the respective functional contributions of endothelial and other CD34^+^ populations in the central nervous system.

Claudin-11 (*Cldn11*/CLDN11,) is a cell adhesion molecule best known for its role in maintaining the tight junctions in myelin sheaths [105]. CLDN11 was first identified in the CP, along with CLDN1 and CLDN2, [62], where it was assumed to contribute to epithelial tight junctions. However herein, CLDN11 was found to be restricted to CP fibroblasts through both immunofluorescent analysis of adult animals (Fig. 6B) as well as in an independent ISH study of fetal mice (Additional file 4: Fig. S4 I, J). The CP stroma is also contiguous with the meninges, which also exhibited robust expression of CLDN11 [106]. Finally, in a recent single cell analysis of the choroid of the eye, *Cldn11* was found to be expressed in (ocular) choroid *Pdgfra*^+^ ‘stromal cells’, rather than epithelium or endothelium [107]. Therefore, the functions of CLDN11 in the CP are likely mediated by the mesenchymal constituents of the stroma rather than secretory CP epithelium.

The first direct link between WNT5A and hindbrain development comes from a recent study which suggested that the CP transports WNT5A to the cerebellum from the CP [64]. Interestingly, while adult CP epithelial cells express high levels of the transcript for the WNT trafficking protein *Wls*, no *Wnt* transcripts were detected. In contrast, *Wnt2* and *Wnt5a* were appreciably detected in the CP-associated mesenchymal cells (Fig. 3C). This may explain the modest CP and cerebellar phenotypes in *Wnt5a* conditional knockouts observed in the *Foxj1-Cre* (epithelium-specific) driven KO model used by Kaiser et al. [64], in comparison to the dramatic morphological changes observed using Nestin-Cre (which labels a variety of cell types in development, likely including embryonic fibroblasts) [108]. Most recently, a study examining the CP itself as a target of WNT5A signaling revealed that complete *Wnt5a* knockout results in catastrophic failure of CP development by ~ E12.5, resulting from deficiencies in planar cell polarity and a failure of the CP to form cohesive epithelial monolayers [109]. This suggests that WNT5A produced from the underlying mesenchymal cells is necessary not only for cerebellar morphogenesis, but also for CP formation. Our observations that *Wnt5a* expression is restricted to the CP mesenchymal cells further necessitates investigation into the role of neurovascular WNT signaling, with this information aiding in the selection of appropriate Cre lines. Within the PG, where *Wnt5a* is also found to be largely restricted to mesenchymal cells and glial cells, it functions within the canonical beta-catenin pathway to mediate PG growth and size [110]. The possibility of mesenchymal cell-derived WNT5A playing a role in BBB permeability via canonical beta-catenin signaling [4] also merits further exploration.

## Conclusion

This study examined the cell-specific transcriptome of fibroblasts and pericytes in the CP and PG, and using optimized conditions for their capture, enabled the comprehensive analysis of these enriched subpopulations. Several novel vascular associated mesenchymal cell subpopulations were identified, along with the identification of distinct markers which will facilitate further analysis in these various compartments. Furthermore, the focus on mesenchymal cells enabled the identification of distinct subpopulations of fibroblasts within the anterior PG and posterior PG. Finally, several genes known to be associated with CP and PG functions were identified to be restricted to the mesenchymal cells that are resident to these regions. In several instances, the cell type expressing these transcripts has evaded analysis. Importantly, this study characterizes mesenchymal cells in the distinct vascular environments of the CP and PG, and provides a molecular framework for better understanding their functional contribution to the unique activities of these tissues.

### Supplementary Information

**Additional file 1: Figure S1.** Pituitary endocrine cell subsets of PG scRNA-Seq. Heat-map of pituitary endocrine cells from scRNA-seq in Figure 4, with differentially expressed genes indicative of somatotrophs, corticotrophs, gonadotrophs, lactotrophs and melanotrophs.

**Additional file 2: Figure S2.** Additional validation of ALPL and CD34 in CP and PG fibroblasts. **A**, **B** 20 × and 60 × magnification images showing the distribution of ALPL and Hic1-tdTom in PG meninges (sagittal section). **C** Chromogenic alkaline phosphatase staining of 4th ventricle CP and PG validates immunofluorescence findings of abundant ALPL in CP fibroblasts and PG meninges. **D** Representative image of CD34 co-localized to Hic1-tdTom in the posterior PG, overlapping regions appear yellow. **E** Representative image of CD34 in anterior PG, where it does not co-localize with Hic1-tdTom.

**Additional file 3: Figure S3.** Astrocyte staining and violin plots of common and novel mesenchymal cell specific genes. **A** The astrocyte marker GFAP is not appreciably expressed in the 4th ventricle CP (sagittal section) and does not co-localize with tdTom^+^ cells in the PG. **B** Violin plots of common systemic markers of mesenchymal cells across aggregated 4^th^ ventricle CP and PG scRNA-seq reveals variable patterns of expression. **C** Violin plots of genes established in CP literature across aggregated 4th ventricle CP and PG scRNA-seq reveals largely specific expression within mesenchymal cells. **D** UMAPs of the pituitary scRNA-seq data that highlights region-specific gene expression within the pituitary mesenchymal cell compartment. Legend coloured by cell type cluster.

**Additional file 4: Figure S4.** Validation of fibroblast-specific genes from external datasets. **A** UMAP of cells collected from rat pineal gland by Mays et al. [45]. **B**, **D** Violin plots of pineal gland with genes specific to mesenchymal cells (*Hic1*, **B**), pinealocytes (*Ddc*, **C**) and astrocytes (*Penk*, **D**). **E**, **F** Violin plots of pineal gland showing expression of *Cldn11 *and *Inmt*, both of which are specific to fibroblasts in the pineal gland. **G**, **H** ISH from Allen Brain Atlas [111–114] showing expression of *Tcf21* and *Inmt* in adult 4th ventricle CP (sagittal section). **I**, **J** ISH from Allen Developing Brain Atlas [111–114] showing *Cldn11* expression at E13.5 and E15.5 in the meninges and 4th ventricle CP (sagittal section).

**Additional file 5: Figure S5.** Re-analysis of published scRNA-seq data of CVOs reveals fibroblast-specific expression of *Inmt. A* UMAP of scRNA-seq data of the OVLT [70] with major cell type clusters identified. **B** UMAP of scRNA-seq data of the SFO [70] with major cell type clusters labelled. **C**, **D** Violin plots of *Hic1,* *Inmt, Htr2c *and *Postn*, showing expression patterns across clusters shown in OVLT (**A**) and SFO (**B**). **E** Representative images of the OVLT from sagittal sections, showing an abundance of tdTom^+^, *Pdgfra*^H2B-EGFP/+^ cells in close proximity to CD31^+^, CD34^+^ vasculature, that were also INMT^+^.

**Additional file 6: Table S1.** Table of primary antibodies.
