# Supplementary figures and images for: Single-cell analysis of mesenchymal cells in permeable neural vasculature reveals novel diverse subpopulations of fibroblasts
Source: Fluids Barriers CNS. 2024 Apr 5;21:31. doi: 10.1186/s12987-024-00535-7 (PMC10996213; doi:10.1186/s12987-024-00535-7)

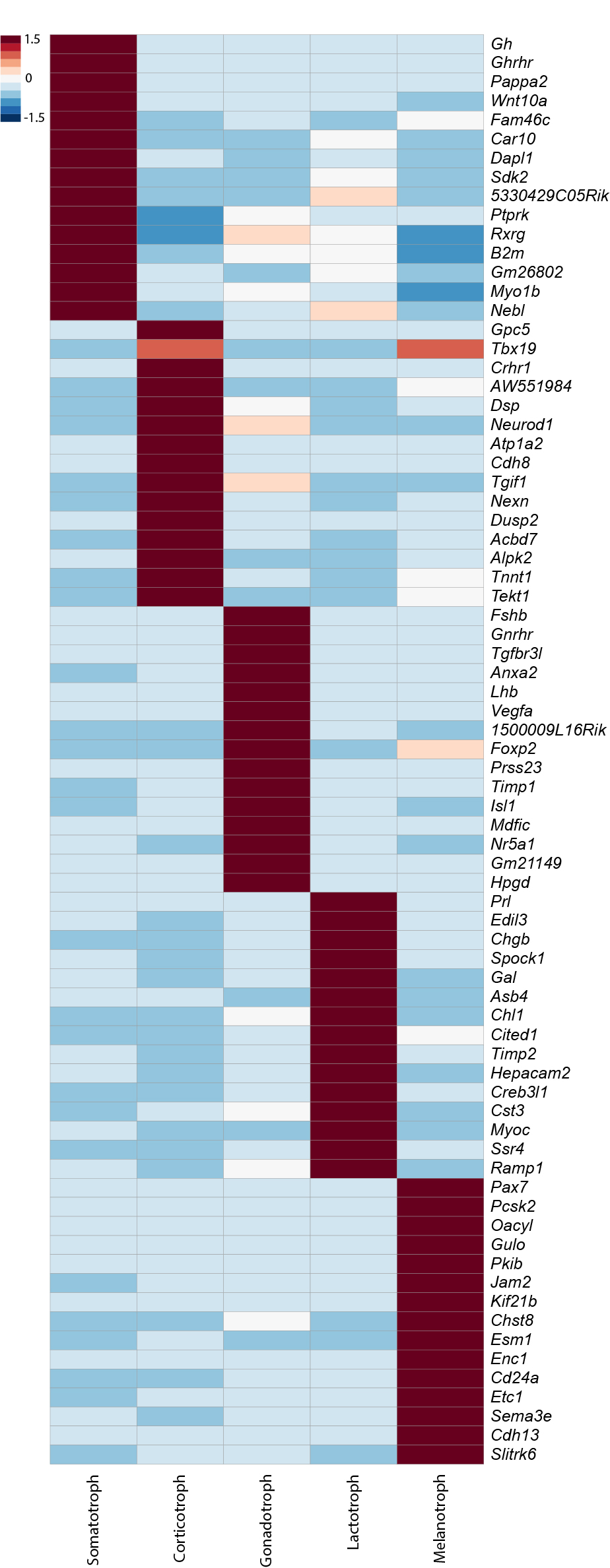

Supplement: Supplementary file 1 — Additional file 1: Figure S1. Pituitary endocrine cell subsets of PG scRNA-Seq. Heat-map of pituitary endocrine cells from scRNA-seq in Figure 4, with differentially expressed genes indicative of somatotrophs, corticotrophs, gonadotrophs, lactotrophs and melanotrophs. [file 12987_2024_535_MOESM1_ESM.jpg]

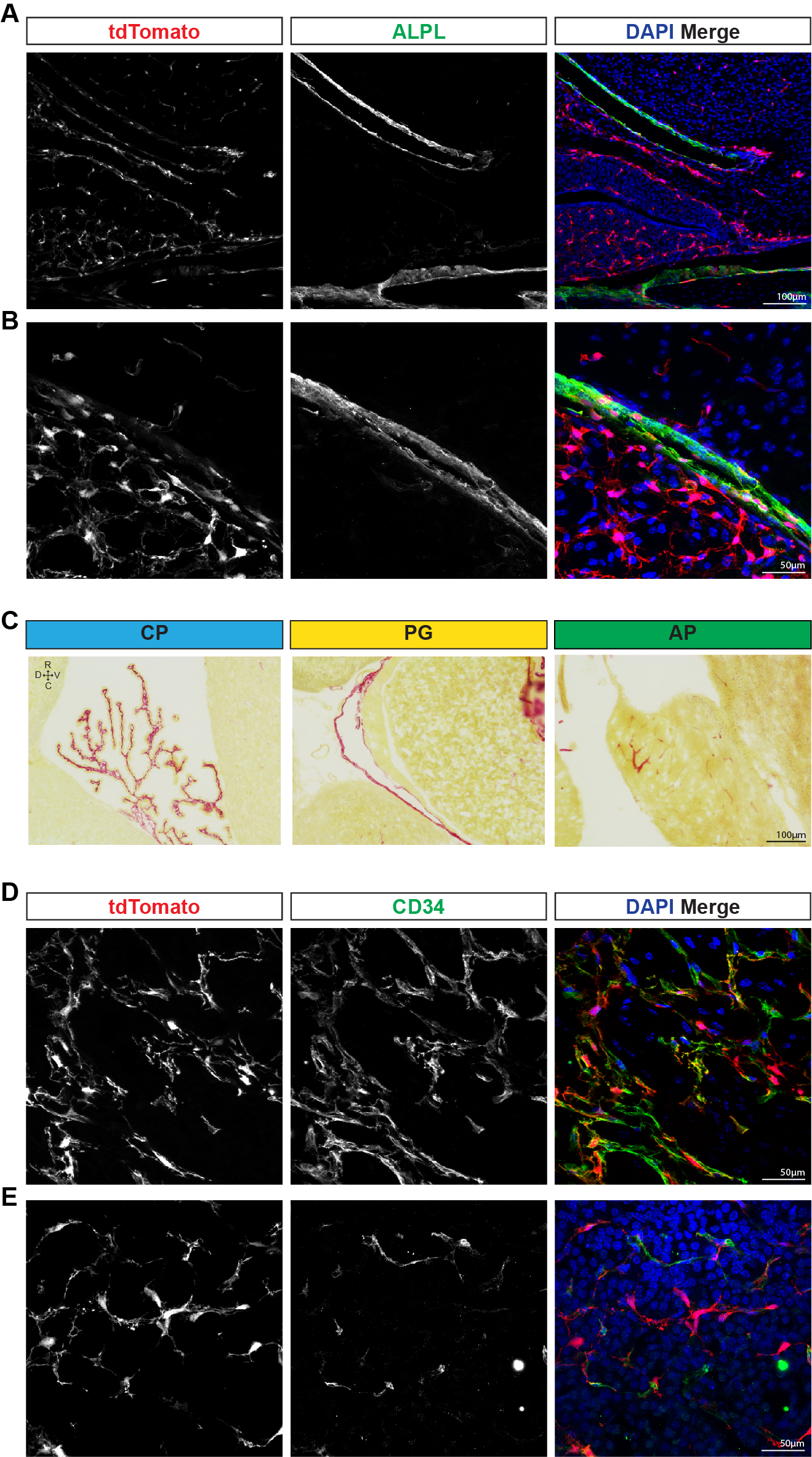

Supplement: Supplementary file 2 — Additional file 2: Figure S2. Additional validation of ALPL and CD34 in CP and PG fibroblasts. A, B 20 × and 60 × magnification images showing the distribution of ALPL and Hic1-tdTom in PG meninges (sagittal section). C Chromogenic alkaline phosphatase staining of 4th ventricle CP and PG validates immunofluorescence findings of abundant ALPL in CP fibroblasts and PG meninges. D Representative image of CD34 co-localized to Hic1-tdTom in the posterior PG, overlapping regions appear yellow. E Representative image of CD34 in anterior PG, where it does not co-localize with Hic1-tdTom. [file 12987_2024_535_MOESM2_ESM.jpg]

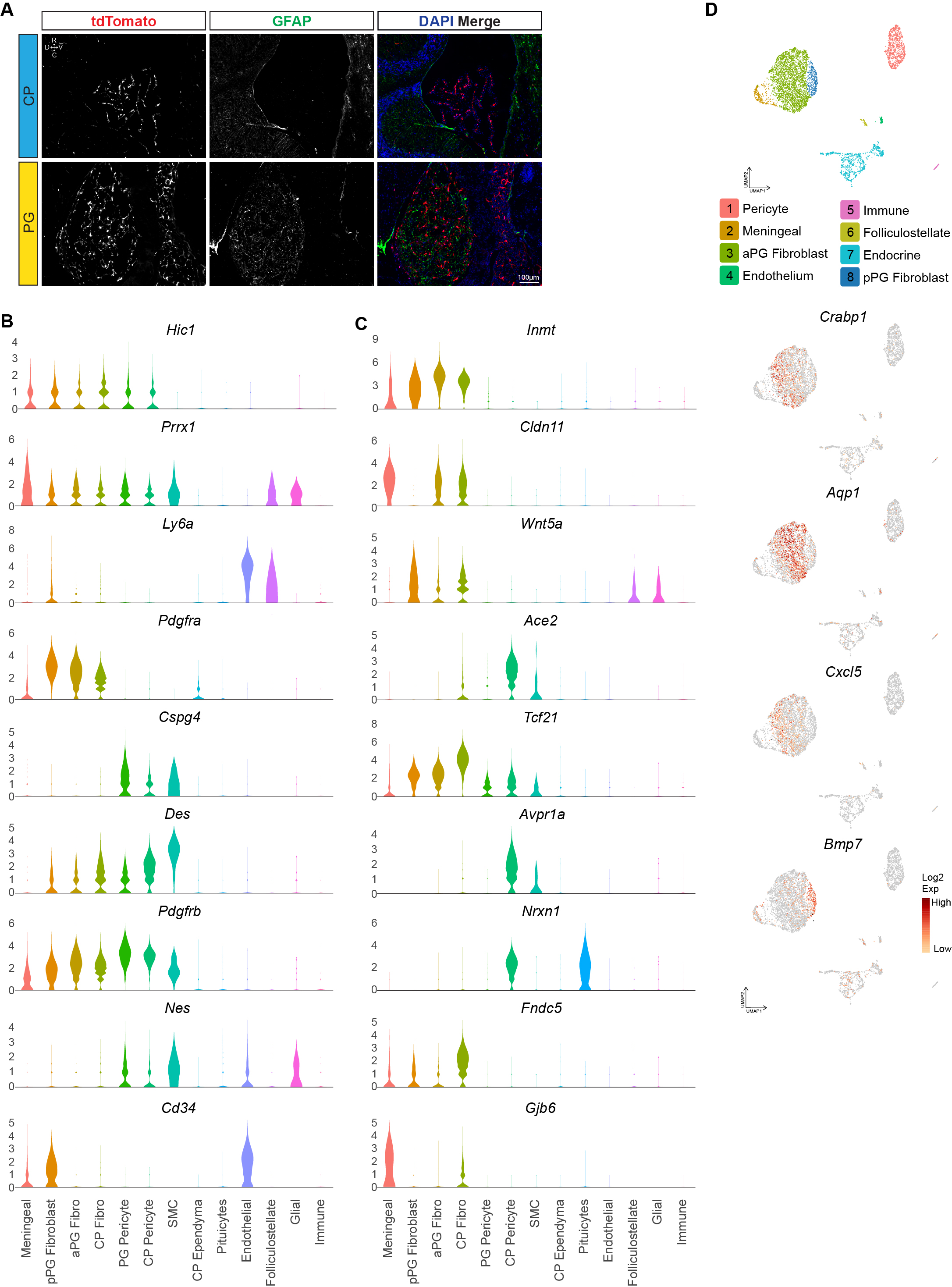

Supplement: Supplementary file 3 — Additional file 3: Figure S3. Astrocyte staining and violin plots of common and novel mesenchymal cell specific genes. A The astrocyte marker GFAP is not appreciably expressed in the 4th ventricle CP (sagittal section) and does not co-localize with tdTom+ cells in the PG. B Violin plots of common systemic markers of mesenchymal cells across aggregated 4th ventricle CP and PG scRNA-seq reveals variable patterns of expression. C Violin plots of genes established in CP literature across aggregated 4th ventricle CP and PG scRNA-seq reveals largely specific expression within mesenchymal cells. D UMAPs of the pituitary scRNA-seq data that highlights region-specific gene expression within the pituitary mesenchymal cell compartment. Legend coloured by cell type cluster. [file 12987_2024_535_MOESM3_ESM.jpg]

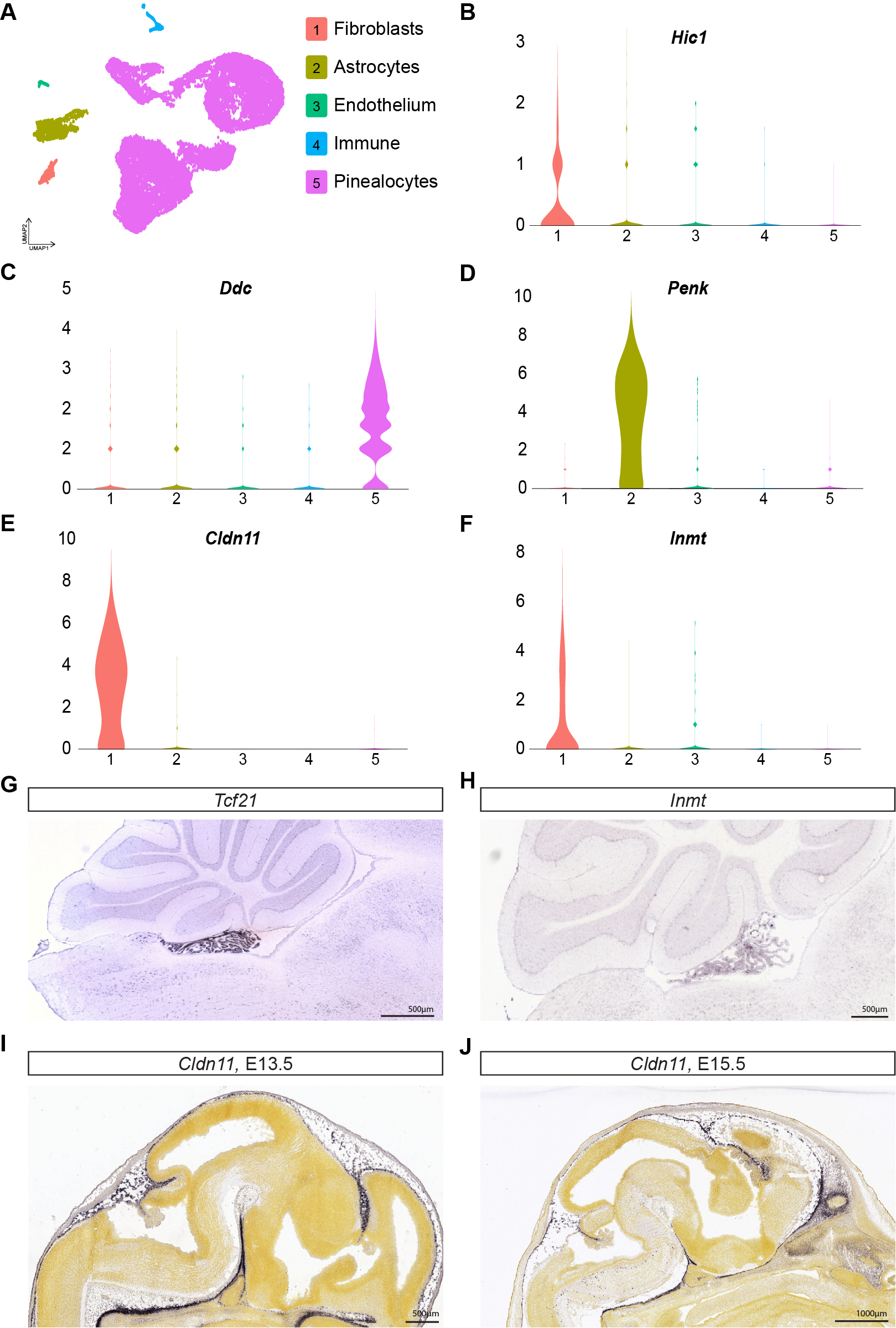

Supplement: Supplementary file 4 — Additional file 4: Figure S4. Validation of fibroblast-specific genes from external datasets. A UMAP of cells collected from rat pineal gland by Mays et al. [45]. B, D Violin plots of pineal gland with genes specific to mesenchymal cells (Hic1, B), pinealocytes (Ddc, C) and astrocytes (Penk, D). E, F Violin plots of pineal gland showing expression of Cldn11 and Inmt, both of which are specific to fibroblasts in the pineal gland. G, H ISH from Allen Brain Atlas [111–114] showing expression of Tcf21 and Inmt in adult 4th ventricle CP (sagittal section). I, J ISH from Allen Developing Brain Atlas [111–114] showing Cldn11 expression at E13.5 and E15.5 in the meninges and 4th ventricle CP (sagittal section). [file 12987_2024_535_MOESM4_ESM.jpg]

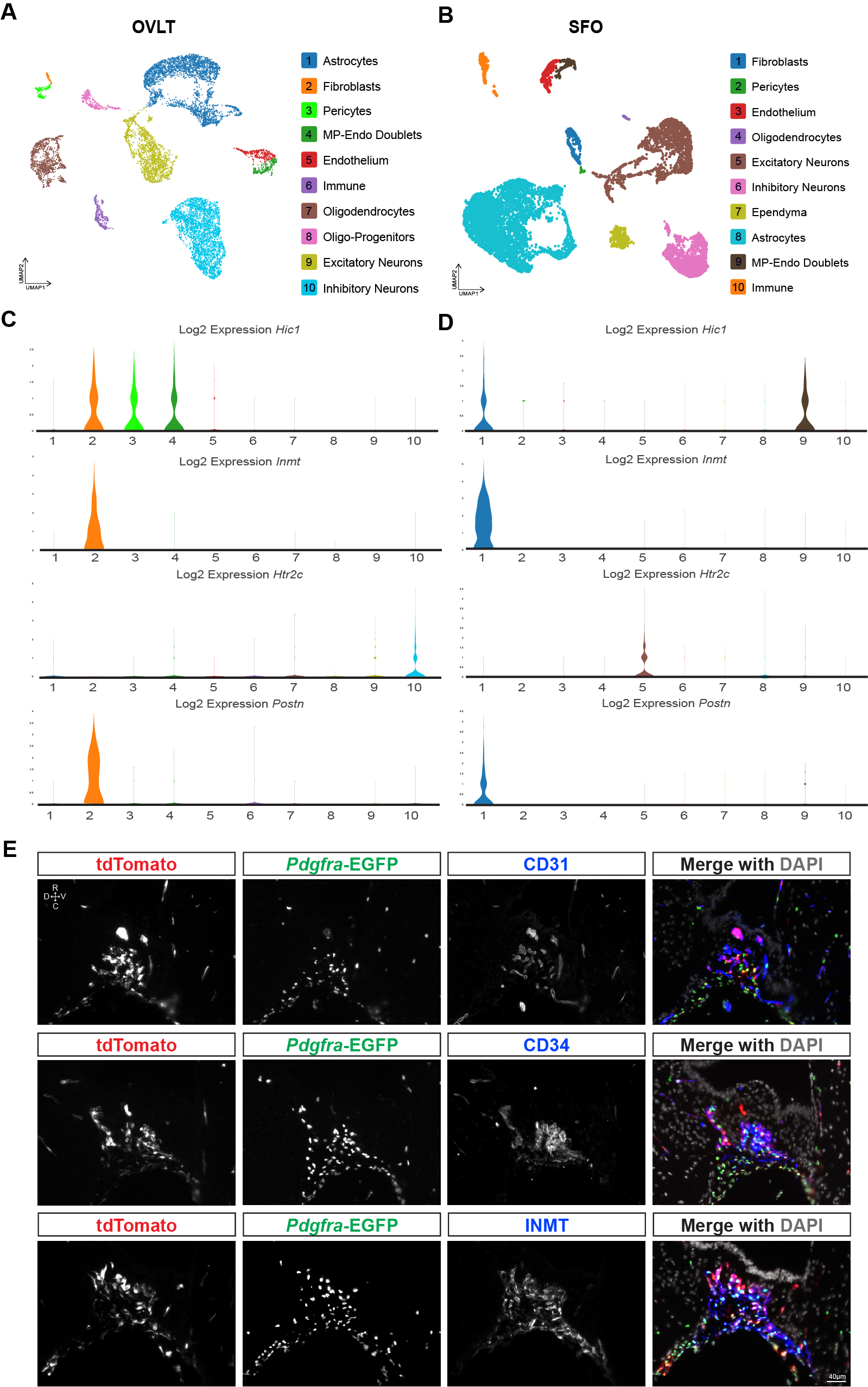

Supplement: Supplementary file 5 — Additional file 5: Figure S5. Re-analysis of published scRNA-seq data of CVOs reveals fibroblast-specific expression of Inmt. A UMAP of scRNA-seq data of the OVLT [70] with major cell type clusters identified. B UMAP of scRNA-seq data of the SFO [70] with major cell type clusters labelled. C, D Violin plots of Hic1, Inmt, Htr2c and Postn, showing expression patterns across clusters shown in OVLT (A) and SFO (B). E Representative images of the OVLT from sagittal sections, showing an abundance of tdTom+, PdgfraH2B-EGFP/+ cells in close proximity to CD31+, CD34+ vasculature, that were also INMT+. [file 12987_2024_535_MOESM5_ESM.jpg]
