# Supplementary material for: Single-cell analysis of mesenchymal cells in permeable neural vasculature reveals novel diverse subpopulations of fibroblasts
Source: Fluids Barriers CNS. 2024 Apr 5;21:31. doi: 10.1186/s12987-024-00535-7 (PMC10996213; doi:10.1186/s12987-024-00535-7)
Supplement: Supplementary file 6 — Additional file 6: Table S1. Table of primary antibodies. [file 12987_2024_535_MOESM6_ESM.docx]

**Table S1.** Table of primary antibodies

| Table S1 List of Primary Antibodies | | | | |
| --- | --- | --- | --- | --- |
| Antigen | Manufacturer | Host | Dilution | Product # |
| Pecam-1 | BD Biosciences | Rat | 1:50 | Cat # 550274 |
| Na/K/ATPase | Abcam | Rabbit | 1:100 | Ab76020 |
| Laminin | Abcam | Rabbit | 1:100 | Ab11575 |
| SOX2 | Abcam | Rabbit | 1:200 | Ab97959 |
| IBA1 | Abcam | Rabbit | 1:100 | Ab178846 |
| TUBB3 | Abcam | Rabbit | 1:100 | Ab18207 |
| Alkaline Phosphatase | R&D Systems | Goat | 1:100 | AF2910 |
| CD34 | Abcam | Rat | 1:100 | Ab8158 |
| ACTA2 | Abcam | Rabbit | 1:200 | Ab5694 |
| CLDN11 | Abcam | Rabbit | 1:100 | Ab53041 |
| INMT | Abcam | Rabbit | 1:100 | Ab181854 |
| LEPR | R&D Systems | Goat | 1:50 | AF497 |
| CD45-647 | UBC Ablab | Rabbit | 1:400 | Cat # 67-0047-01 |
| CD31-APC | BD Biosciences | Rabbit | 1:400 | Cat # 551262 |
| Ter119-647 | UBC Ablab | Rabbit | 1:400 | Cat # 67-0031-01 |
| Alexa Fluor 488 anti-rabbit IgG | ThermoFisher | Goat | 1:500 | Cat # A11034 |
| Alexa Fluor 647 anti-rabbit IgG | ThermoFisher | Goat | 1:500 | Cat # A21245 |
| Alexa Fluor 647 anti-rat IgG | ThermoFisher | Goat | 1:500 | Cat # A21247 |
| Alexa Fluor 488 anti-goat IgG | Abcam | Donkey | 1:500 | Ab150129 |
| Alexa Fluor 647 anti-mouse IgG | ThermoFisher | Goat | 1:500 | Cat # A11029 |
